# Histone acetylation promotes long-lasting defense responses and longevity following early life heat stress

**DOI:** 10.1371/journal.pgen.1008122

**Published:** 2019-04-29

**Authors:** Lei Zhou, Bin He, Jianhui Deng, Shanshan Pang, Haiqing Tang

**Affiliations:** School of Life Sciences, Chongqing University, Chongqing, China; University of Massachusetts Medical School, UNITED STATES

## Abstract

Early exposure to some mild stresses can slow down the aging process and extend lifespan, raising the question of how early life stress might impact the somatic health of aged animals. Here, we reveal that early life heat experience triggers the establishment of epigenetic memory in soma, which promotes long-lasting stress responses and longevity in *C*. *elegans*. Unlike lethal heat shock, mild heat activates a unique transcriptional program mimicking pathogen defense responses, characterized by the enhanced expression of innate immune and detoxification genes. Surprisingly, the expression of defense response genes persists long after heat exposure, conferring enhanced stress resistance even in aged animals. Further studies identify the histone acetyltransferase CBP-1 and the chromatin remodeling SWI/SNF complex as epigenetic modulators of the long-lasting defense responses. Histone acetylation is elevated by heat stress and maintained into agedness thereafter. Accordingly, histone acetylation levels were increased on the promoters of defense genes. Moreover, disruption of epigenetic memory abrogates the longevity response to early hormetic heat stress, indicating that long-lasting defense responses are crucial for the survival of aged animals. Together, our findings provide mechanistic insights into how temperature stress experienced in early life provides animals with lifetime health benefits.

## Author summary

Organism aging is a deleterious process characterized by the progressive decline of cellular and tissue functions in the late life stage. However, the rate of aging is often determined by an organism’s historic experiences that occur in early life. For example, some mild stresses experienced in early life can extend animal lifespan, implying that early stresses might impact the health of aged animals, the mechanisms of which remain largely unknown. Here, by using the model organism *C*. *elegans*, we reveal a mechanism of how early life heat stress impacts the health of aged animals and promotes longevity. We find that early exposure to mild heat activates basal innate immune and detoxification responses, which are surprisingly maintained long after temperature drop, even into agedness, and therefore contribute to lifespan extension. Remarkably, the long-lasting defense responses require epigenetic memory established by histone acetyltransferase CBP-1 and chromatin remodeling complex SWI/SNF. As a previous study has reported high temperature-induced transgenerational epigenetic effects in germline that involve histone methylation, our findings suggest that temperature may trigger distinct epigenetic changes in soma and germline, conferring adaptations in both the parental generation and their offspring.

## Introduction

Early life events pose significant impacts on the health of adults, even the elderly. For example, the aging process, characterized by the decline of tissue function in late life, is often determined by the historic experiences of organisms in early life or even in the parental generations, yet the underlying mechanisms are only beginning to be uncovered in model organisms. Studies in nematode *C*. *elegans* show that mitochondrial stress induced during development can promote longevity via modulation of histone methylation [1,2]. Additionally, transgenerational inheritance of chromatin modulations in germline regulated by H3K4me3-modifying enzymes could increase *C*. *elegans* lifespan, even for generations [3–5]. Multiple types of environmental stresses, such as temperature and starvation, induce long-lasting epigenetic changes in the *C*. *elegans* germline and lead to inheritable phenotypes for generations [6–9]. However, whether these environmental stresses also trigger epigenetic memory in somatic tissues and thus cause long-lasting phenotypes into agedness needs further exploration.

Animals are continuously confronted with various stresses that have substantial effects on the aging process. Therefore, animals have evolved delicate mechanisms to defend against various detrimental stresses and ensure long-term health and longevity. Such mechanisms involve stress responses that facilitate resistance to multiple harmful stresses, including unfolded proteins, reactive oxygen species, pathogens, toxic chemicals, etc. Notably, whereas severe stresses are detrimental to animal survival, exposure to mild stresses is often beneficial for health and longevity, a phenomenon referred to as hormesis. Enhanced stress resistance in response to mild stimuli is believed to defend against aging-related cellular damage and therefore promote somatic maintenance [10]. More importantly, early life exposure to mild environmental stress is often sufficient to slow down the aging process and extend lifespan [11–14], raising the intriguing question of how stress resistance elicited early in life might affect the health of aged animals and set the rate of aging.

Temperature is an important environmental factor with substantial impacts on animal physiology and aging. Lower environmental temperature promotes longevity in poikilotherms, such as worms and flies [15–17]. Additionally, homeothermal rodents with lower core temperatures exhibit longer lifespan [18], implying that lifespan regulation by temperature might be evolutionarily conserved. On the contrary, higher ambient temperature, representing mild heat stress, is harmful to animals and shortens lifespan [19–21]. Further study has discovered that high environmental temperature has a hormetic effect on aging, as *C*. *elegans* exposed to high temperature only during development exhibited an extended lifespan [20]. In addition, early life exposure to acute heat shock, the extremely high and lethal temperature, also increases lifespan [11,12,22], implying that shared mechanisms may be utilized by the two distinct temperature sets. These studies suggest that temperature might have long-lasting effects on animal physiology. As high temperature-induced transgenerational epigenetic effects in germline have been observed in *C*. *elegans* [6,8,9], the somatic epigenetic program might also memorize the temperature information, which, together with germline epigenetic memory, confers adaptations in both the parental generation and their offspring.

In this study, we focused on animal responses to high environmental temperature in soma and identified mechanisms linking early life heat stress to the health of aged animals. We first analyzed the transcriptome of *C*. *elegans* at high temperature and revealed PMK-1-dependent innate immune and detoxification responses as essential longevity mechanisms to mild heat. Further, we uncovered that early life heat stress established epigenetic memory in *C*. *elegans*, which was maintained into adulthood and led to long-lasting activation of defense responses and therefore promoted somatic maintenance and longevity in aged animals. The key epigenetic mechanism we revealed was histone acetylation that increases on the promoters of defense response genes. Collectively, our findings show how early life environmental stress establishes epigenetic memory in soma and promotes persistent stress responses, which may have life-long health benefits.

## Results

### High temperature activates innate immune and detoxification responses

**Fig 1:**
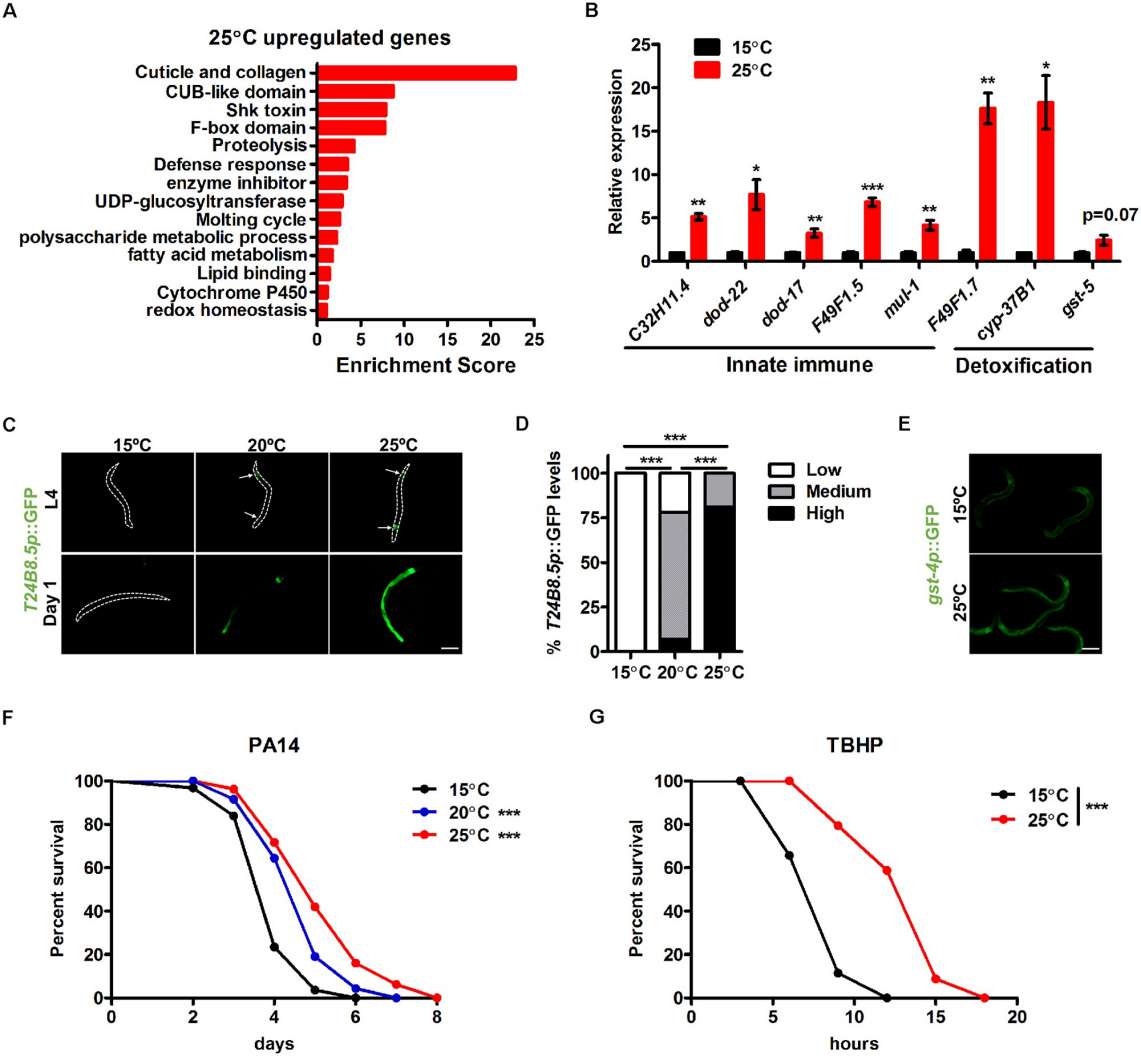
High environmental temperature activates innate immune and detoxification responses. (A) DAVID functional annotation analysis of 25°C-upregulated genes. (B) Expression of innate immune and detoxification genes analyzed by qPCR. n = 3 for each group. Data are presented as mean ± SEM. Benjamini-Hochberg adjusted p value * < 0.05, ** < 0.01, *** < 0.001 versus 15°C controls. (C-D) Representative images (C) and quantification of GFP levels (D) of *T24B8*.*5p*::GFP in L4 and day 1 adult worms cultivated under different temperatures. Arrows indicate GFP signals. The GFP levels were scored as low, medium and high. Number of biological replicates (n): 15°C (89), 20°C (86) and 25°C (95). ***p < 0.001 via Chi-square and Fisher’s exact tests. (E) Expression of *gst-4p*::GFP at 15°C and 25°C. (F) Pathogen resistance of worms precultivated at 15°C, 20°C and 25°C. PA14: *P*. *aeruginosa* strain PA14. ***p < 0.001 versus 15°C controls via log-rank test. (G) Survival of worms precultivated at 15°C and 25°C upon tert-butyl hydroperoxide (TBHP) treatment.***p < 0.001 versus 15°C controls via log-rank test. Scale bar = 100 μm.

The nematode *C*. *elegans* is a powerful genetic model for studying temperature effects at the organismal level. In the laboratory, *C*. *elegans* is cultivated at physiological temperatures ranging from 15°C to 25°C. Cultivation at 25°C high temperature throughout life shortens *C*. *elegans* lifespan, whereas 25°C exposure only during development promotes longevity [20]. As stress resistance is often believed to confer longevity in response to multiple hormetic stresses, we first sought to understand the activation of stress responses at high temperature by comparing the transcriptome of 25°C-cultivated animals to that of 15°C-cultivated control animals through RNA-seq analysis. A total of 1958 transcripts were differentially regulated, including 1143 that were upregulated and 815 that were downregulated (S1 Table). Functional annotation analysis revealed that genes activated by 25°C were enriched in the following categories: cuticle development, antimicrobial function, proteolysis and xenobiotic metabolism (Fig 1A; S1 Table), implying their roles in temperature adaptation.

Notably, many genes associated with pathogen stress responses were induced by high temperature. For example, the upregulation of antimicrobial and xenobiotic genes indicates the activation of innate immune and detoxification responses, respectively, both of which are implicated in the modulation of pathogen defense as well as the aging process. A recent transcriptome study in *C*. *elegans* also revealed the activation of innate immune genes by high temperature [23]. As such, we analyzed the related genes involved in these two responses and their physiological consequences in detail. Functional analysis showed that the enriched antimicrobial genes were CUB-like genes and Shk toxins (Fig 1A, S1 Table). Xenobiotic genes enriched were UDP-glucuronosyltransferases (UGTs) and cytochrome P450 family members (CYPs) (Fig 1A, S1 Table). In addition, five glutathione S-transferases (GSTs) genes, another group of xenobiotic genes, also exhibited higher expression levels at 25°C. The number of GST genes that were induced by high temperature was greater than that expected by random chance (one would expect less than 3 GST genes by random chance). We further confirmed the expression of several innate immune and detoxification genes by qPCR (Fig 1B).

We next examined the expression of well-established transcriptional immune reporters for *T24B8*.*5* and *F35E12*.*5* [24,25] and the xenobiotic reporter *gst-4p*::GFP [26] in live animals. Cultivation at 25°C from L1 to L4 larvae induced modest *T24B8*.*5p*::GFP expression, and more robust GFP activation was observed when cultivation continued to day 1 adults (Fig 1C and 1D). The endogenous *T24B8*.*5* mRNA levels were elevated over 100-fold by high temperature (S1A Fig). Similarly, reporters *F35E12*.*5p*::GFP (S1B Fig) and *gst-4p*::GFP (Fig 1E) were also activated by 25°C cultivation.

Next, we evaluated whether the increased expression of pathogen-responsive genes conferred enhanced stress resistance by employing the *C*. *elegans—P*. *aeruginosa* host-pathogen system. We found that *C*. *elegans* cultivated at 25°C from L1 larvae to day 1 adults indeed exhibited enhanced pathogen resistance compared to worms cultured at 15°C (Fig 1F; S2 Table). In addition, because xenobiotic metabolism detoxifies harmful products during infection and aging, e.g., bacterial toxins and endogenous reactive oxygen species (ROS), we further examined animal resistance to oxidative stress and observed an increased survival rate of high temperature-cultivated worms (Fig 1G; S3 Table). These data suggest that high temperature activates innate immune and detoxification responses to promote animal stress resistance.

Since worms were fed with relatively non-pathogenic *E*. *coli* OP50, it is possible that high temperature increases the bacterial growth rate and gut colonization, which might be sensed by hosts, thus activating the immune and detoxification responses. We ruled out this possibility by showing that 25°C precultivated *C*. *elegans* fed on dead OP50 still exhibited activation of the immune reporter *T24B8*.*5p*::GFP (S1C, S1D and S1E Fig) and a higher survival rate upon pathogen infection (S1F Fig; S2 Table). Thus, the activation of stress responses may result from direct sensing of temperature changes rather than the gut bacteria.

### PMK-1 is required for stress resistance and longevity at high temperature

**Fig 2:**
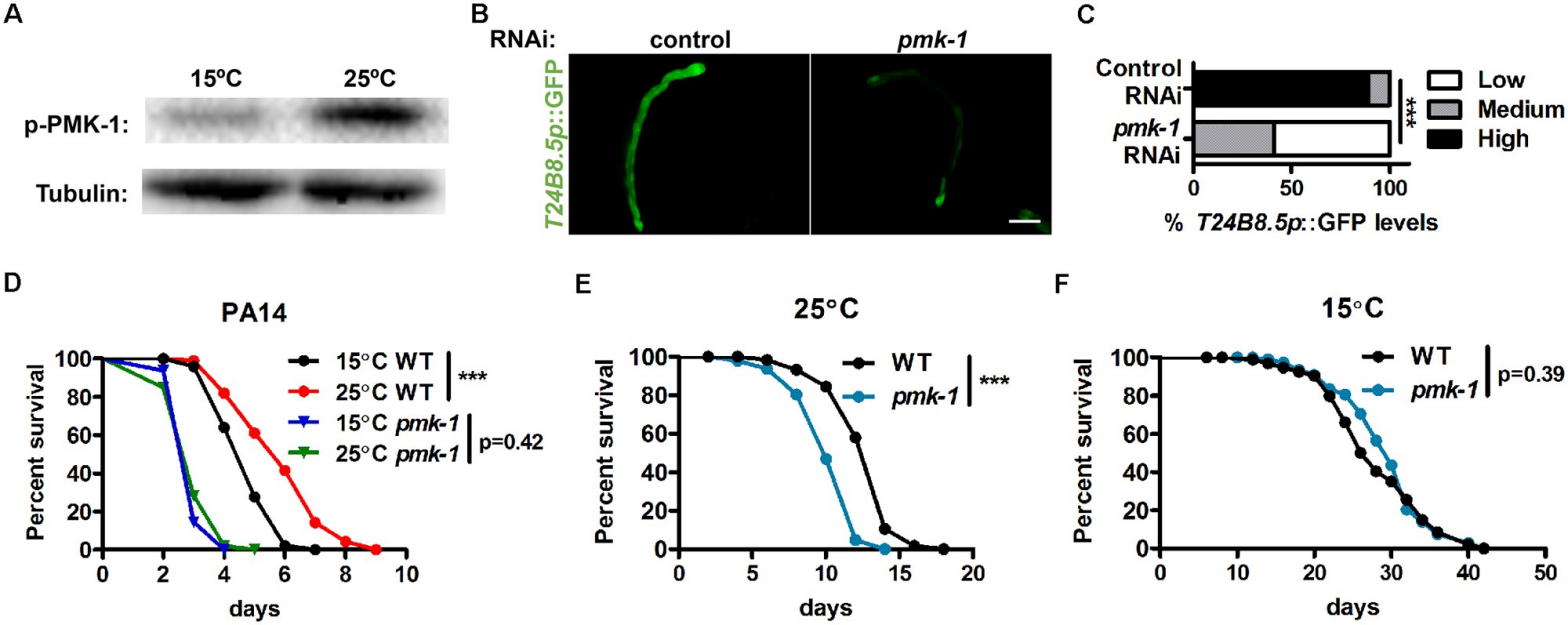
High temperature activates defense responses via PMK-1. (A) PMK-1 activation at 15°C and 25°C as measured by immunoblot analysis. (B) Effects of *pmk-1* RNAi on *T24B8*.*5p*::GFP expression at 25°C. (C) Quantification of GFP levels. Number of biological replicates (n): control RNAi (80) and *pmk-1* RNAi (93). ***p < 0.001 via Chi-square and Fisher’s exact tests. (D) Pathogen resistance of wild-type (WT) and *pmk-1* mutant worms precultivated at 15°C and 25°C. ***p < 0.001 versus 15°C controls via log-rank test. (E-F) Lifespan of WT and *pmk-1* mutants cultivated at 25°C (E) and 15°C (F). ***p < 0.001 versus WT controls via log-rank test. Scale bar = 100 μm.

Conserved PMK-1/p38 mitogen-activated protein kinase is a major component of the *C*. *elegans* immune defense system, which regulates the expression of innate immune and detoxification genes [27,28]. As such, we asked whether PMK-1 mediated defense responses in response to 25°C high temperature. We first examined the activation of PMK-1 at different temperatures and found that PMK-1 phosphorylation levels were significantly increased by high temperature (Fig 2A). In addition, the activation of the innate immune reporter *T24B8*.*5p*::GFP was largely dependent on PMK-1, as *pmk-1* RNAi inhibited its expression at 25°C (Fig 2B and 2C). The mRNA levels of endogenous *T24B8*.*5* and two other defense response genes were further confirmed by qPCR analysis (S2A Fig). Accordingly, mutation of *pmk-1* almost completely abolished the effects of temperature on pathogen resistance (Fig 2D; S2 Table). Together, these findings suggest that high temperature activates the PMK-1-dependent defense response.

Since activation of innate immune and detoxification responses promotes longevity in *C*. *elegans*, we further examined whether PMK-1 regulated longevity at high temperature. If so, the loss function of *pmk-1* would specifically shorten the lifespan of worms cultured at 25°C. As expected, mutation of *pmk-1* indeed significantly compromised the lifespan of animals cultured at 25°C during entire life (Fig 2E; S4 Table), whereas it exhibited no effects at 15°C (Fig 2F; S4 Table). Similar results were also observed when *pmk-1* mutants were fed with dead bacteria (S2B and S2C Fig; S4 Table). Thus, PMK-1 is essential for longevity at 25°C high temperature, likely through the activation of innate immune and detoxification responses.

### Early life heat stress induces persistent stress responses

**Fig 3:**
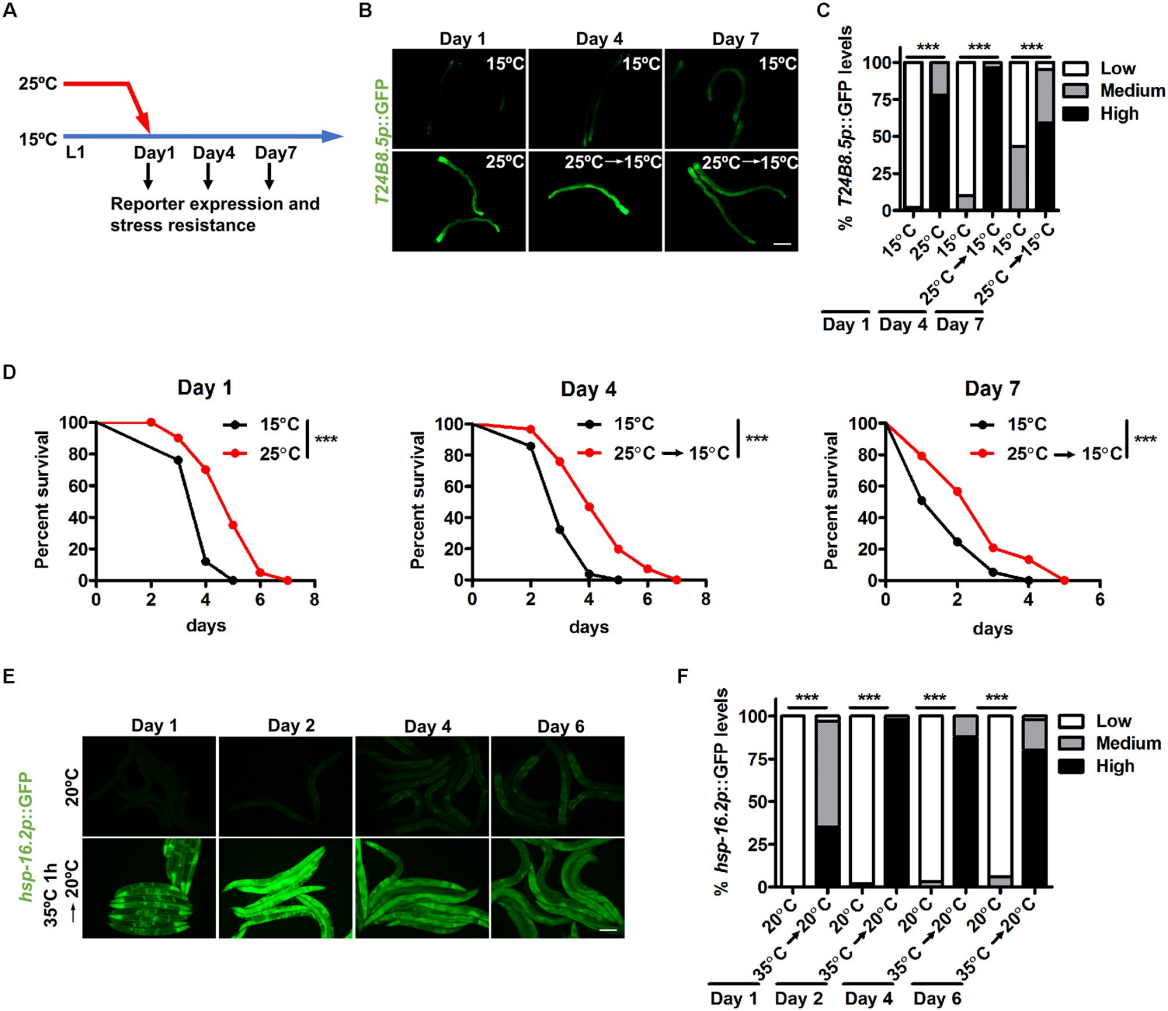
Early life exposure to high temperature promotes persistent stress responses. (A) Schematic of the analysis of stress responses after temperature shift. (B) Worms were cultivated at 25°C until day 1 adults and transferred to 15°C thereafter. *T24B8*.*5p*::GFP expression was examined as indicated in 3A. (C) Quantification of GFP levels. Number of biological replicates (n): 15°C day 1 (98), 25°C day 1 (124), 15°C day 4 (103), 25°C to 15°C day 4 (124), 15°C day 7 (102) and 25°C to 15°C day 7 (107). ***p < 0.001 via Chi-square and Fisher’s exact tests. (D) Pathogen resistance measured as indicated in Fig 3A. ***p < 0.001 versus 15°C controls via log-rank test. (E) Day 1 adult worms were heat shocked at 35°C for one hour and transferred to 20°C thereafter. The expression of *hsp16*.*2p*::GFP was measured as indicated. (F) Quantification of GFP levels. Number of biological replicates (n): 20°C day 1 (111), 35°C day 1 (102), 20°C day 2 (99), 35°C to 20°C day 2 (97), 20°C day 4 (121), 35°C to 20°C day 4 (114), 20°C day 6 (127) and 35°C to 20°C day 6 (106). ***p < 0.001 via Chi-square and Fisher’s exact tests. Scale bar = 100 μm.

Cultivation at mild heat of 25°C during development promotes longevity in *C*. *elegans* [20], implying long-term effects of early life heat on the health of older animals. Considering the significant impacts of stress resistance on longevity regulation, we speculated that early exposure to 25°C might have long-lasting effects on somatic stress responses. To test this, worms were cultivated at 25°C until day 1 adults and then shifted to 15°C. The expression of GFP reporters for stress responses was then examined during the remainder of their lives (Fig 3A). As indicated in Fig 3A, day 4 represents midlife, and day 7 represents agedness. Remarkably, we observed that animals exposed to 25°C until day 1 adults exhibited persistently elevated expression of the immune reporter *T24B8*.*5p*::GFP on day 4 and day 7 when compared to controls kept at 15°C from the L1 larval stage, with higher expression levels on day 4 than day 7 (Fig 3B and 3C). Another immune reporter, *F35E12*.*5p*::GFP, as well as the xenobiotic reporter *gst-4p*::GFP, also showed similar expression patterns (S3A and S3B Fig). In addition, qPCR analysis confirmed the persistent mRNA induction of defense genes, including *T24B8*.*5*, *F49F1*.*7* and *cyp-37B1* (S3C Fig). Moreover, animal resistance to pathogen and oxidative stresses were also maintained (Fig 3D and S3D Fig; S2 and S3 Tables), representing health benefits in aged animals. Taken together, we conclude that early exposure to high temperature is sufficient to promote long-lasting stress resistance.

Heat shock temperature induces an adaptive response characterized by increased expression of heat shock proteins. We examined whether the heat shock response induces a similar long-lasting phenotype. We examined the expression of *hsp-16*.*2p*::GFP, a well-known reporter for heat shock response, and observed its dramatic activation after one hour exposure to 35°C on day 1 as previously reported [29] (Fig 3E and 3F). Surprisingly, worms continuously exhibited strong activation of *hsp-16*.*2p*::GFP even six days after heat-shock recovery at 20°C (Fig 3E and 3F). We also observed a similar expression pattern of endogenous *hsp-16*.*2* mRNA by qPCR (S3E Fig). Consistently, animals showed persistently enhanced heat shock resistance (S3F Fig; S3 Table), indicating that heat shock could also trigger constant activation of stress response. Taken together, we propose that animals could memorize the effects of hormetic heat stress in soma via the long-lasting activation of stress response genes, which may provide long-term protection against aging-related stresses.

### CBP-1 mediates the activation of defense responses to heat stress

What are the mechanisms underlying the persistent enhancement of stress resistance? Epigenetic regulations are usually involved in long-term effects of environmental factors on animal physiology [30]. We thus speculated that specific epigenetic factors might be responsible for the observed persistent phenotype. We performed a small-scale RNAi screening targeting putative *C*. *elegans* epigenetic genes (S5 Table) [31–33], using 25°C *T24B8*.*5p*::GFP activity as a readout, and two genes *cbp-1* and *swsn-1* were identified. CBP-1, ortholog of the mammalian CBP protein, is a histone acetyltransferase that plays an essential role in lifespan extension in dietary restricted *C*. *elegans* [34]. However, how CBP-1 regulates lifespan and the relationship between CBP-1 and defense response genes remain largely elusive.

**Fig 4:**
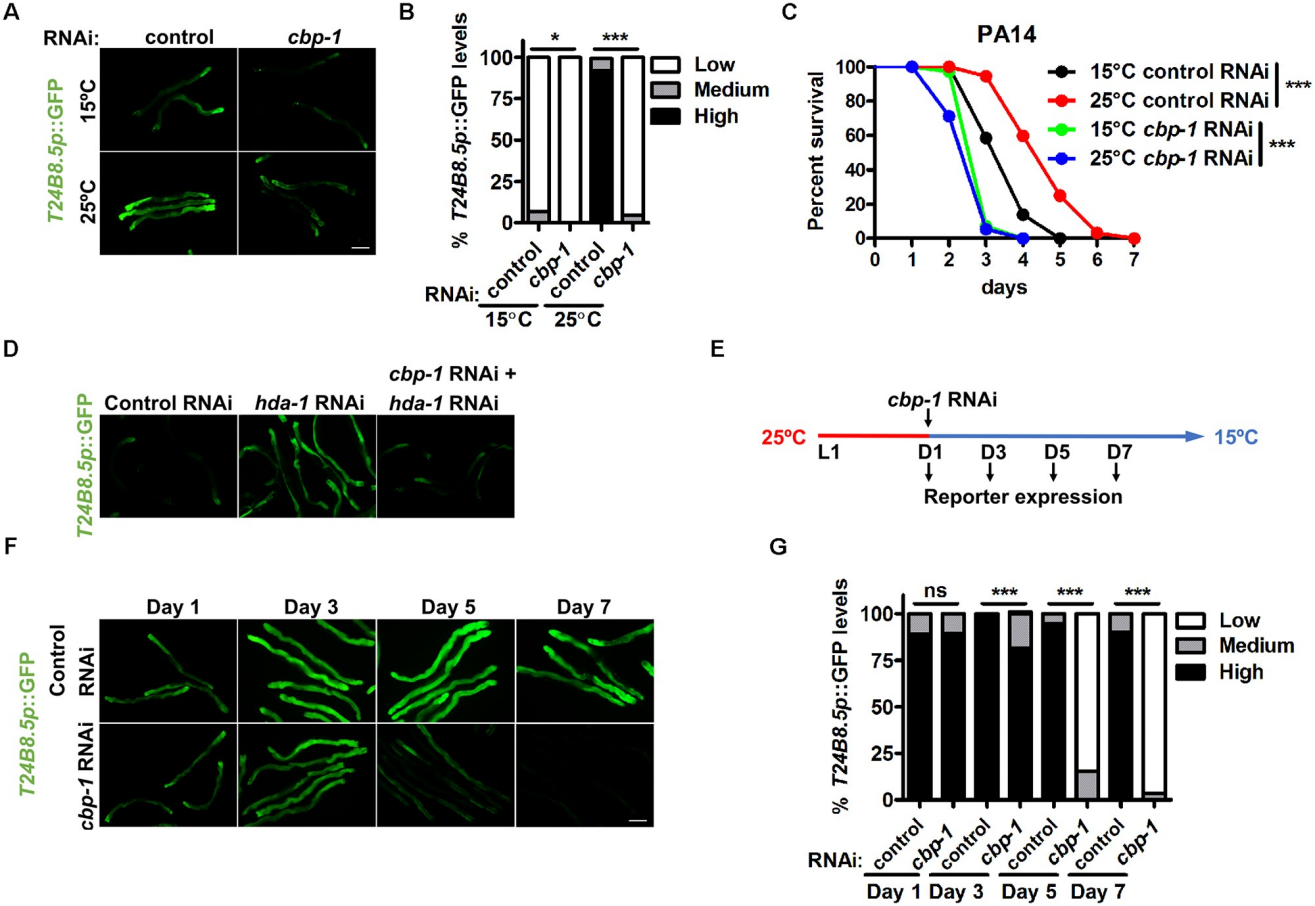
CBP-1 is required for high temperature-induced stress responses. (A) Effects of *cbp-1* RNAi on *T24B8*.*5p*::GFP expression at different temperatures. (B) Quantification of GFP levels. Number of biological replicates (n): control RNAi 15°C (67), *cbp-1* RNAi 15°C (79), control RNAi 25°C (84) and *cbp-1* RNAi 25°C (91). *p < 0.05, ***p < 0.001 via Chi-square and Fisher’s exact tests. (C) Effects of *cbp-1* RNAi on pathogen resistance. ***p < 0.001 versus 15°C RNAi via log-rank test. (D) Effects of RNAi targeting *hda-1* and *cbp-1* on *T24B8*.*5p*::GFP expression at 15°C. (E) Schematic of analysis of stress reporter upon postdevelopmental treatment of *cbp-1* RNAi. (F) Expression of *T24B8*.*5p*::GFP measured as indicated in 4E. (G) Quantification of GFP levels. Number of biological replicates (n): control RNAi day 1 (91), *cbp-1* RNAi day 1 (80), control RNAi day 3 (65), *cbp-1* RNAi day 3 (72), control RNAi day 5 (87), *cbp-1* RNAi day 5 (79), control RNAi day 7 (88) and *cbp-1* RNAi day 7 (94). *** p < 0.001 via Chi-square and Fisher’s exact tests. Scale bar = 100 μm.

Because *cbp-1* RNAi suppressed worm development, we used 20-fold diluted *cbp-1* RNAi to ensure normal development of worms and verified the effect of diluted RNAi on knockdown of *cbp-1* expression (S4A Fig). We found that RNAi of *cbp-1* almost completely abolished the activation of *T24B8*.*5p*::GFP (Fig 4A and 4B), as well as the expression of endogenous *T24B8*.*5*, *F49F7*.*1* and *C32H11*.*4* (S4B Fig) at 25°C. The enhancement of pathogen resistance at 25°C was also abrogated by *cbp-1* RNAi (Fig 4C; S2 Table). Importantly, the effects of CBP-1 on the expression of defense response genes were specific, as knockdown of other histone acetyltransferases in *C*. *elegans* was unable to suppress *T24B8*.*5p*::GFP expression at 25°C (S4C Fig). Furthermore, 25°C culture failed to promote *gst-4p*::GFP activity (S4D Fig) and oxidative stress resistance (S4E Fig; S3 Table) once *cbp-1* was knocked down, indicating critical roles for CBP-1 in stress resistance at high temperature. Histone acetylation levels are regulated by both histone acetyltransferase and deacetylase. Therefore, inhibition of histone deacetylase would increase histone acetylation. We next examined whether activation of histone acetylation was sufficient to induce defense response genes at lower temperatures by using RNAi targeting several histone deacetylase genes in *C*. *elegans* (S5 Table) [31]. The results showed that knockdown of *hda-1*, which encodes histone deacetylase 1, was indeed sufficient to induce *T24B8*.*5p*::GFP at 15°C (Fig 4D) that could be suppressed by *cbp-1* RNAi treatment (Fig 4D). These data further support that the expression of defense response genes is regulated by histone acetylation.

We next assessed the specificity of *cbp-1* RNAi in the regulation of stress response genes. CBP-1 has been found to mediate the stress gene activation and longevity response to dietary restriction in *C*. *elegans* [34]. Consistent with previous findings, *cbp-1* RNAi indeed inhibited the induction of detoxification genes, including *gst-7* and *cyp-37B1*, in dietary restriction model *eat-2* mutants (S4F Fig) [35]. Intriguingly, the induction of *gst-5*, another detoxification gene, did not require CBP-1 in *eat-2* worms, suggesting that *cbp-1* RNAi does not suppress gene induction in general. Mutation of insulin/IGF-1 receptor *daf-2* increases *C*. *elegans* lifespan that requires stress response transcription factor DAF-16 and SKN-1 [36,37]. Of note, in *daf-2* mutants, *cbp-1* RNAi had either slight or no effects on the induction of the DAF-16 target gene *sod-3* and the SKN-1 target genes, *gst-5* and *gst-10* (S4G Fig). Lack of obvious effects of *cbp-1* RNAi on the induction of the DAF-16 and SKN-1 target genes was also observed in germline-deficient *glp-1* mutants (S4H Fig), another well-known longevity model in *C*. *elegans* [38]. Collectively, these data suggest that the effects of CBP-1 on stress gene expression are not nonspecific but depend on context.

### CBP-1 mediates the persistent activation of stress responses

We next asked whether CBP-1 accounted for the long-lasted activation of stress response genes. We reasoned that if CBP-1 was involved in this process, then *cbp-1* knockdown after heat exposure would abolish the maintenance of *T24B8*.*5p*::GFP activation. We conducted *cbp-1* RNAi treatment on heat-exposed day 1 adult animals (Fig 4E) that already exhibited strong induction of *T24B8*.*5p*::GFP. As expected, *T24B8*.*5p*::GFP expression started to diminish two days after *cbp-1* RNAi treatment (day 3), became much lower on day 5 and was completely undetectable on day 7, while control RNAi worms still showed strong and persistent activation of *T24B8*.*5p*::GFP (Fig 4F and 4G). The expression pattern of endogenous *T24B8*.*5* was also verified by qPCR (S4I Fig). We thus conclude that high temperature promotes long-lasting activation of stress responses via CBP-1.

### Histone acetylation regulates the expression of defense response genes at high temperature

**Fig 5:**
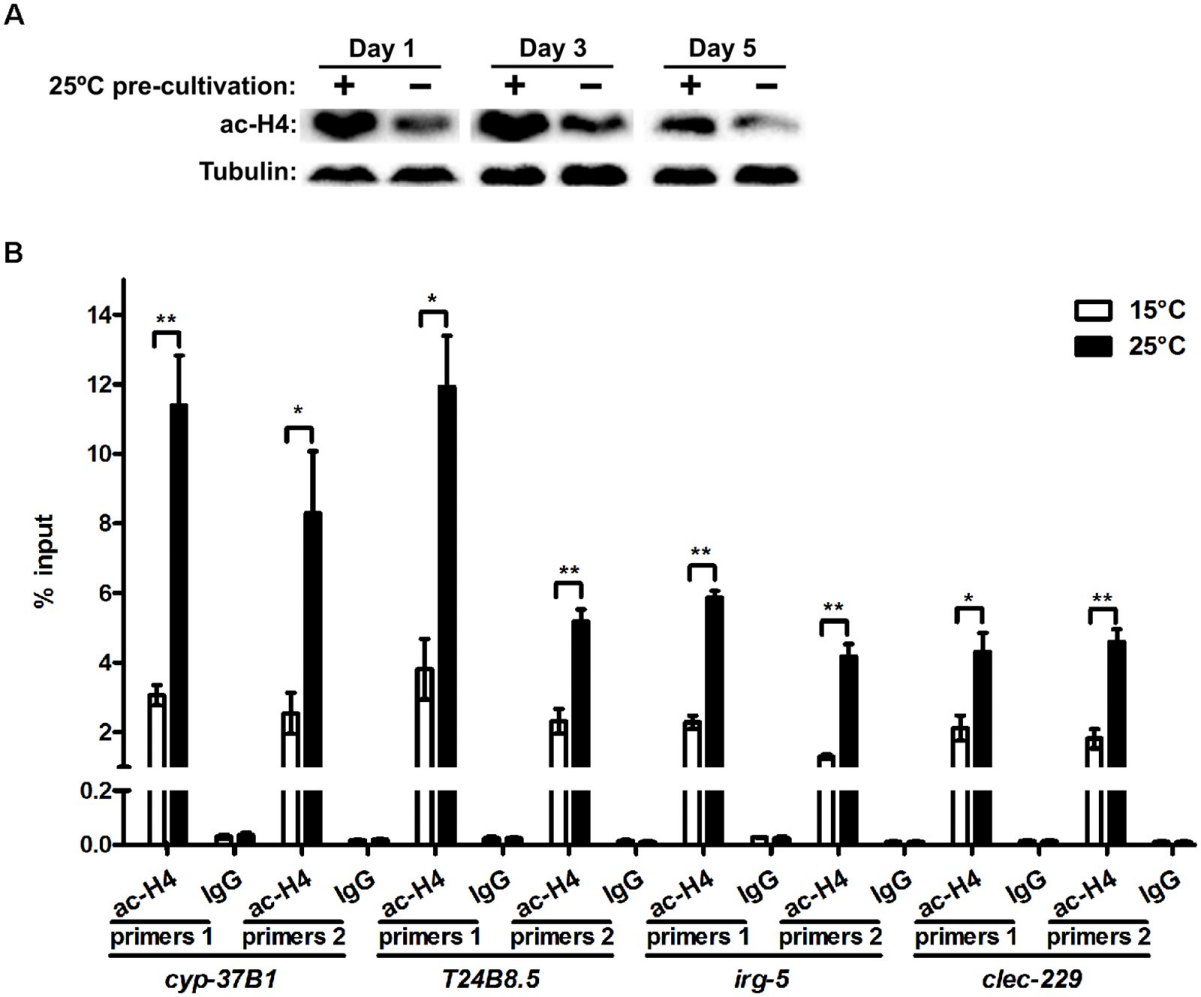
Histone acetylation regulates defense response genes at high temperature. (A) Worms were cultivated at 25°C until day 1 adults and were then transferred to 15°C. Acetylated H4 levels were measured on day 1, day 3 and day 5 by western blot. (B) ChIP-qPCR results in worms cultivated at 15°C and 25°C by using an anti-acetyl-histone H4 antibody. Anti-IgG antibody was used as a negative control. n = 3 for each group. Data are presented as mean ± SEM. Benjamini-Hochberg adjusted p value * < 0.05, ** < 0.01.

Increased histone acetylation is typically associated with enhanced gene expression. The crucial role for CBP-1 in temperature-induced activation of defense response genes implies the involvement of histone acetylation. Consistent with the enhanced expression of stress response genes, we found that histone acetylation, indicated by acetylated H4 levels, was dramatically increased at 25°C (Fig 5A), which was abrogated by *cbp-1* RNAi (S5A Fig). Because PMK-1 is a critical upstream molecule regulating defense response genes at high temperature (Fig 2), we next checked the regulation of histone acetylation by PMK-1. As expected, the *pmk-1* mutation abolished the induction of histone acetylation at 25°C (S5B Fig), suggesting that high temperature promotes histone acetylation through PMK-1.

As CBP-1 mediated the persistent activation of defense response genes (Fig 4F and 4G), histone acetylation might also be maintained. Indeed, we observed persistent elevation of acetylated H4 levels after 25°C cultivation (Fig 5A). To further explore whether temperature regulates the expression of defense genes directly through histone acetylation, we examined the occupancy of acetylated histone on the promoter regions of defense response genes by ChIP-qPCR analysis (S5C Fig, the schematic of promoter regions detected by qPCR). We chose four defense response genes with great induction at 25°C based on RNA-seq results. The results revealed 2- to 4-fold increases of acetylated H4 levels on the promoters of the four genes at 25°C (Fig 5B). In contrast, no changes of H4 acetylation were observed on the promoters of *acdh-4* and *pde-4* (S5D Fig), two randomly selected genes whose expression was not affected by temperature based on RNA-seq analysis (S5E Fig). Together, these data suggest that high temperature might promote the activation of defense response genes by increasing histone acetylation at their promoters.

### SWSN-1 modulates stress resistance at high temperature

**Fig 6:**
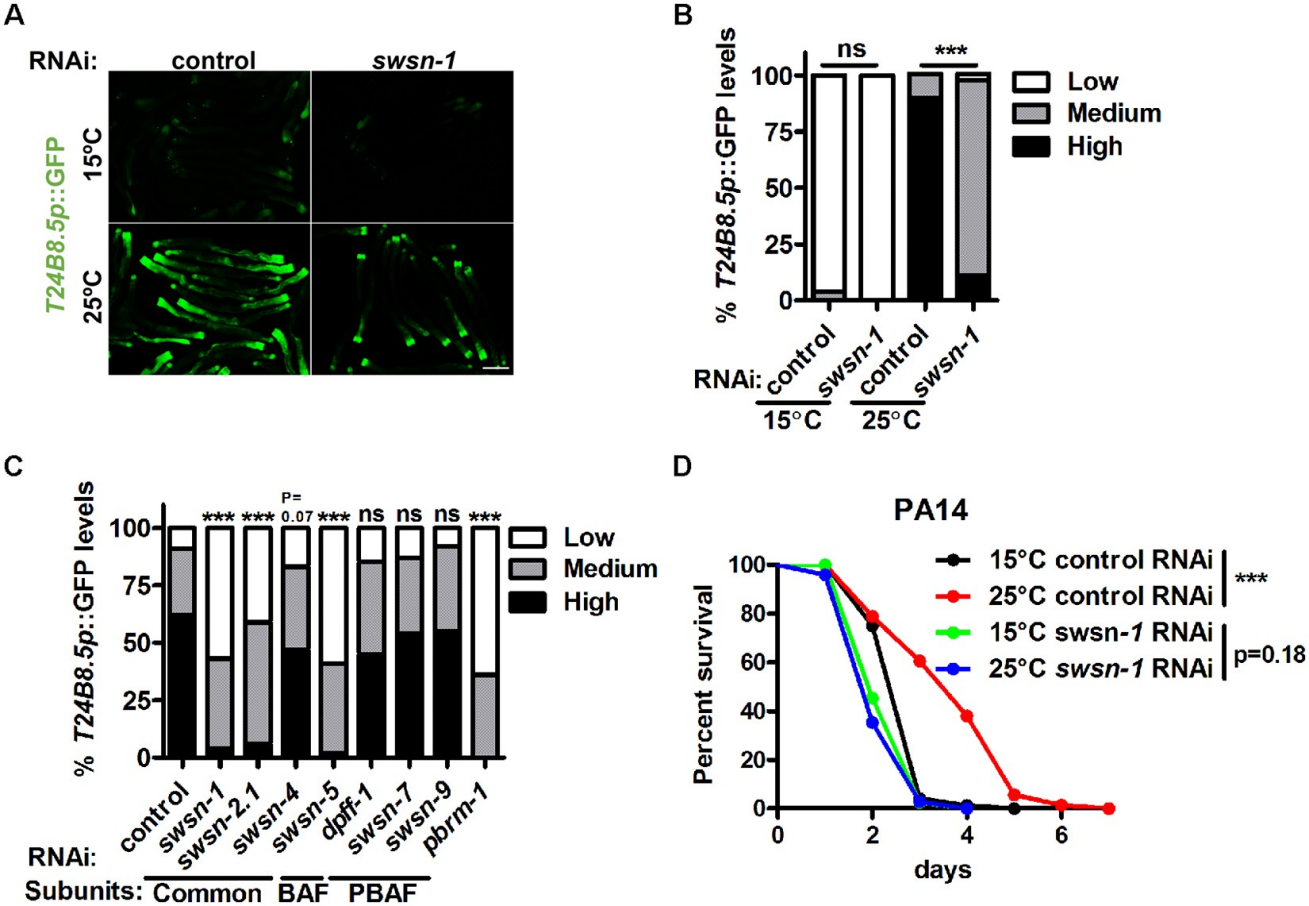
SWSN-1 is required for high temperature-induced stress responses. (A) Effects of *swsn-1* RNAi on the expression of *T24B8*.*5p*::GFP. (B) Quantification of GFP levels. Number of biological replicates (n): control RNAi 15°C (86), *swsn-1* RNAi 15°C (75), control RNAi 25°C (73) and *swsn-1* RNAi 25°C (70). ***p < 0.001 via Chi-square and Fisher’s exact tests. (C) Expression of *T24B8*.*5p*::GFP levels upon treatment of RNAi targeting components of the SWI/SNF complex at 25°C. Number of biological replicates (n): control RNAi (65), *swsn-1* RNAi (74), *swsn-2*.*1* RNAi (70), *swsn-4* RNAi (69), s*wsn-5* RNAi (68), *dpff-1* RNAi (78), *swsn-7* RNAi (66), *swsn-9* RNAi (97) and *pbrm-1* RNAi (86). ***p < 0.001 versus control RNAi via Chi-square and Fisher’s exact tests. (D) Effects of *swsn-1* RNAi on the pathogen resistance of worms precultivated at 15°C and 25°C. ***p < 0.001 versus 15°C RNAi via log-rank test. Scale bar = 100 μm.

SWSN-1 is another epigenetic regulator of *T24B8*.*5p*::GFP identified at 25°C (Fig 6A and 6B), and the activation of the xenobiotic reporter *gst-4p*::GFP was also suppressed by *swsn-1* RNAi (S6A Fig). *swsn-1* encodes the subunit of the SWI/SNF complex, an important chromatin remodeling apparatus that displaces or exchanges nucleosomes to open chromatin [39–41]. The SWI/SNF complex has been reported to regulate lifespan as a cofactor of DAF-16; however, its function has not yet been linked to temperature or defense responses. The SWI/SNF complex is composed of common and accessory subunits. Two subclasses of SWI/SNF, PBAF and BAF, are defined by their accessory signature subunits (S6B Fig). The *C*. *elegans* genome encodes thirteen subunits of the SWI/SNF complex, among which eight genes have corresponding RNAi clones in the *C*. *elegans* Ahringer RNAi collection (S6B Fig). By knocking down the eight subunit genes, we found that three of them, including the common subunits *swsn-2*.*1* and *swsn-5* and the PBAF signature subunit *pbrm-1*, also participated in *T24B8*.*5p*::GFP regulation at 25°C (Fig 6C), further supporting that the SWI/SNF complex, possibly the PBAF subclass, play important roles in mediating high temperature-induced stress responses.

Next, we examined the effects of the SWI/SNF complex on stress resistance regulation by using RNAi targeting the common subunit gene *swsn-1*. Knockdown of *swsn-1* completely abolished 25°C-induced resistance to pathogen and oxidative stress (Fig 6D and S6C Fig; S2 and S3 Tables). These data implicate that the chromatin remolding complex SWI/SNF function as another epigenetic regulator of stress responses to high temperature.

### Somatic epigenetic memory promotes longevity in response to hormetic high temperature

**Fig 7:**
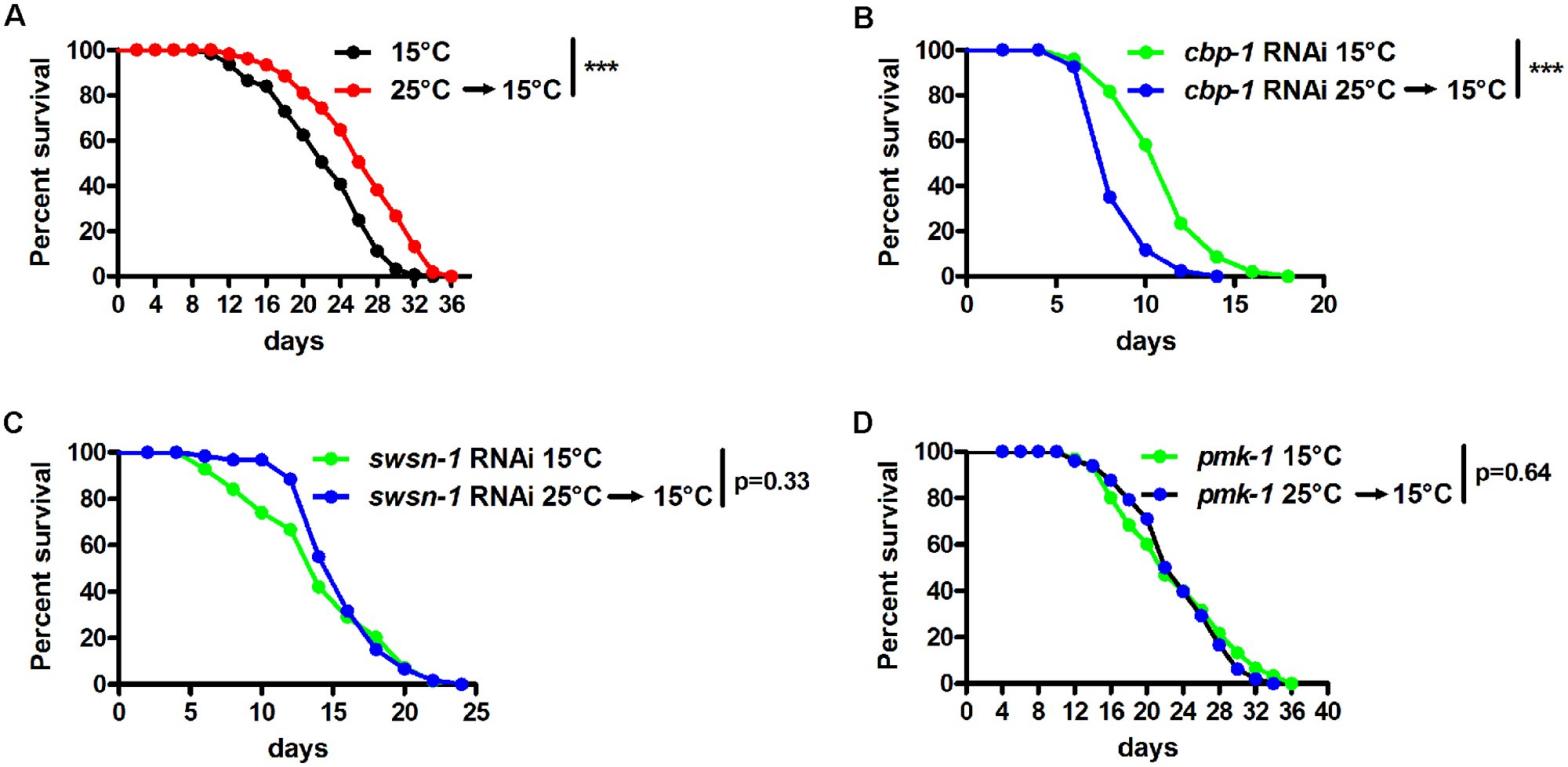
Crucial regulators of longevity in response to early hormetic heat stress. (A) Developmental exposure to 25°C extended *C*. *elegans* lifespan. (B-C) Effects of *cbp-1* (B) and *swsn-1* (C) RNAi on the longevity of worms exposed to 25°C during development. Worms were fed on RNAi bacteria during entire life. (D) Effects of *pmk-1* mutation on the lifespan of worms exposed to 25°C during development. ***p < 0.001 versus 15°C controls via log-rank test.

Early life 25°C heat stress exhibits hormetic effects that extend lifespan in *C*. *elegans* [20]. Our findings imply an intriguing explanation for this phenotype, that the induction of persistent defense responses protects animals against aging-related damage such as endogenous ROS and infiltrated bacteria. If so, disruption of long-lasting defense responses, by silencing genes of the upstream somatic epigenetic program, would abolish the longevity response to hormetic heat stress. To test the hypothesis, we used RNAi or mutation to silence *cbp-1*, *swsn-1* and *pmk-1* individually in worms and examined their lifespan in response to early life heat stress. As previously reported [20], 25°C cultivation from L1 larvae to day 1 adults extended animal lifespan (Fig 7A; S4 Table). Remarkably, knockdown of *cbp-1* completely abrogated the beneficial effect of hormetic heat stress on lifespan (Fig 7B; S4 Table) and *swsn-1* RNAi partially abrogated the lifespan extension (Fig 7C; S4 Table). It should be noted that RNAi of *cbp-1* and *swsn-1* significantly shortened lifespan at 15°C. *cbp-1* RNAi was demonstrated to reduce lifespan by accelerating aging rather than causing general sickness [34]. Therefore, it is likely that the same CBP-1-mediated epigenetic mechanisms that support longevity upon heat stress also function to maintain normal lifespan at 15°C. The SWI/SNF complex is a regulator of DAF-16-mediated gene expression [42]. Thus, *swsn-1* RNAi might regulate lifespan at 15°C by affecting DAF-16 target genes. The mutation of *pmk-1*, the crucial regulator of defense response and histone acetylation at 25°C, also completely abolished the lifespan extending effects of hormetic heat stress (Fig 7D; S4 Table). These data support a model in which persistent defense responses, promoted by PMK-1, CBP-1 and SWSN-1, confer health benefits and longevity to aged animals, which were exposed to mild heat stress during development.

## Discussion

Considerable efforts have been devoted to elucidating the lifespan extending effects of hormetic heat stress. The unresolved question is how short-term or transient stress exposure would have long-term health benefits to the organism. We uncovered the persistent activation of defense responses upon mild heat, conferring long-lasting protection against various aging-related stresses. Thus, the organism aging rate is set upon exposure to high temperature in early life, primarily due to the reset of basal activities of essential stress response pathways. The identification of CBP-1 and SWSN-1 as essential regulators further suggests a key role for epigenetic memory in heat adaptation. Knockdown of *cbp-1* after heat exposure was able to suppress the maintenance of stress response gene (Fig 4F), suggesting that CBP-1-mediated epigenetic memory is responsible for the observed persistent gene expression. In addition, the inhibition of CBP-1 and SWSN-1 not only disrupts the establishment and maintenance of stress resistance but also compromises longevity induced by hormetic high temperature, supporting the prevailing view that epigenetic alteration is a hallmark of aging [43,44].

The well-recognized adaptation to temperature rise is the heat shock response. High environmental temperature, however, is different from heat shock and represents a physiological condition, under which organisms can survive and reproduce normally. We identified the innate immune and detoxification responses as major adaptations to high temperature. Since intensive metabolic alterations are induced by temperature rise, elevation of toxic metabolites or byproducts, such as ROS, are probably inevitable, making the activation of xenobiotic metabolism rational. However, why do organisms elevate their basal innate immunity? What are the benefits? In nature, pathogen infectivity is tightly associated with environmental temperature rise. Most bacterial pathogens multiply vigorously and exhibit increased virulence at higher environmental temperatures [45,46]. An intriguing speculation is that environmental temperature rise is interpreted as a signal indicative of high infection risk that resets the organisms’ basal immunity to cope with upcoming infections. Whether the regulation of basal immunity by environmental temperature is evolutionarily conserved has yet to be determined.

We identified the histone acetyltransferase CBP-1 and the chromatin remodeling SWI/SNF complex as epigenetic regulators at high temperature. How are CBP-1 and SWI/SNF regulated by high temperature? They could be transcriptionally upregulated, but our RNA-seq data ruled out this possibility (S1 Table), or they may be recruited by specific transcription factors to act as coactivators [47]. Intriguingly, both CBP-1 and SWSN-1 have been reported as coactivators of the longevity factor DAF-16/FOXO [34,42,48]. Identification of transcription factors that might recruit CBP-1 and the SWI/SNF complex in response to heat stress would be important. Alternatively, histone acetylation could be regulated through the substrate acetyl-CoA. Metabolic pathways producing acetyl-CoA have been implicated as important regulators of histone acetylation [49–51]. Indeed, our RNA-seq results do reveal alterations of many metabolic genes, including those regulating acetyl-CoA levels and histone acetylation in diverse biological processes, yet further studies are needed to test their involvement. In addition, since histone acetyltransferase complexes can stabilize SWI/SNF binding to promoters [52], and the binding of SWI/SNF to particular promoters requires histone acetylation [53], SWI/SNF may be recruited in a CBP-1-dependent manner and function together with histone acetylation to regulate target genes.

In summary, we have identified innate immune and detoxification responses as the major stress responses at high temperature and provided a molecular basis for how stress responses are established and maintained. Our findings provide mechanistic insights into how early life heat stress exerts long-term effects on animal health and sets the rate of organism aging. We propose that persistent activation of stress responses could be a shared adaptive mechanism underlying the longevity response to multiple hormetic stresses.

## Materials and methods

### *C*. *elegans* strains and maintenance

*C*. *elegans* were cultured on standard nematode growth medium (NGM) seeded with *E*. *coli* OP50-1 [54] and maintained at different temperatures as indicated. The following strains were provided by the Caenorhabditis Genome Center: wild type N2 Bristol, KU25[*pmk-1(km25)*], AU78[*T24B8*.*5p*::*gfp*::unc-54-3' UTR], AY101[*F35E12*.*5p*::*gfp*], CL2166[*gst-4p*::*gfp*], TJ375[*hsp-16*.*2p*::*gfp*], DA465[*eat-2(ad465)*], CB4037[*glp-1(e2141)*], DR1572[*daf-2(e1368)*].

### Microbe strains

*E*. *coli* OP50-1 bacteria were cultured overnight at 37°C in LB, after which 150 μl of bacterial culture was seeded on 60 mm NGM plates. For the dead OP50-1 experiment, *E*. *coli* was killed by UV irradiation (254 nm, 10 minutes), heat (75°C, 90 minutes) or kanamycin (400 μg/mL). The death of bacteria was confirmed by streaking bacteria onto LB plates and verifying the lack of colony formation after 37°C culture for 48 hours. For the RNAi experiment, HT115 bacteria containing specific dsRNA-expression plasmids (Ahringer library) [55] were cultured overnight at 37°C in LB containing 100 μg/ml carbenicillin and seeded on NGM plates containing 5 mM IPTG [56]. The pathogen resistance assay was performed as previously described [57]. *P*. *aeruginosa* strain PA14 was cultured overnight at 37°C in LB and seeded on slow killing (SK) plates, then incubated for 24 hours at 37°C followed by another 24 hours at 25°C.

### Stress resistance assays

For the pathogen resistance assay, day 1 adult worms, unless otherwise indicated, were transferred to SK plates and incubated at 25°C for survival analysis. For TBHP (Sigma) resistance, day 1 adult worms, unless otherwise indicated, were transferred to NGM plates supplemented with 12.6 mM TBHP and incubated at 20°C for survival analysis. For heat shock resistance, worms at the indicated stages were transferred to NGM plates and incubated at 35°C for survival analysis.

### RNA interference treatment

RNAi was induced at room temperature for 24 hours after seeding. Then, L1 worms were added to RNAi plates to knockdown the indicated genes. For *cbp-1* RNAi treatment, the bacterial culture of the *cbp-1* RNAi strain was diluted at a ratio of 1:20 with the vector control.

### qRT-PCR

qRT-PCR was performed as previously described [58]. Briefly, worms cultured at 15°C and 25°C until the L4 stages were collected, washed in M9 buffer and then homogenized in TRIzol reagent (Life Technologies). RNA was extracted according to the manufacturer’s protocol. DNA contamination was digested with DNase I (Thermo Fisher Scientific), and RNA was subsequently reverse-transcribed to cDNA by using the RevertAid First Strand cDNA Synthesis Kit (Thermo Fisher Scientific). Quantitative PCR was performed using SYBR Green (Bio-Rad). The expression of *snb-1* was used to normalize samples. Primer sequences are listed in S6 Table.

### Fluorescence microscopy

Day 1 adult worms, unless otherwise indicated, were collected, washed in M9 buffer and then paralyzed with 1 mM levamisole. Fluorescence microscopic images were taken after mounting on slides. The expression levels of GFP were scored as low, medium and high. Briefly, dim green signals in the anterior or posterior of the intestine, green signals throughout the intestine and bright signals throughout the intestine are categorized as low, medium and high expression, respectively.

### Immunoblotting

Day 1 adult worms, unless otherwise indicated, were collected and sonicated in RIPA buffer (100 mM Tris pH 8.0, 150 mM NaCl, 1% Triton X-100, 1% deoxycholic acid, 0.1% SDS, 5 mM EDTA, 10 mM NaF) containing 1 mM DTT and proteinase inhibitor (Sigma) before boiling and loading. Antibodies against acetyl-histone H4 (Millipore), phospho-p38 (Promega) and tubulin (Sigma) were used.

### Lifespan analysis

Lifespan assays were performed as previously described [59] with modifications. Briefly, synchronized eggs were added to nematode growth medium (NGM) plates seeded with different *E*. *coli* strains. For experiments with developmental exposure to 25°C, worms were kept at 25°C from the egg to L4 stage and then shifted to 15°C thereafter. Worms were transferred every day during the reproductive period. Worms that died of vulva burst, bagging, or crawling off the plates were censored.

### RNA-seq analysis

To compare the effects of temperatures on gene expression, 20°C-cultured L4 parent worms were moved to 15°C and 25°C separately, and allowed to mature further and lay eggs, which hatched and developed into middle-to-late L4 stages at these temperatures. The 15°C-cultured parent worms were allowed to lay eggs about 40 hours earlier than the 25°C-cultured ones, making the two groups developmentally equivalent across temperatures. Total RNA from 15°C and 25°C-cultivated L4 stage worms was extracted using TRIzol reagent and used to generate sequencing libraries using the VAHTS Stranded mRNA-seq Library Prep Kit for Illumina. The quality and quantity of the libraries were determined using an Agilent Bioanalyzer 2100 (Agilent, USA) and Qubit 3.0 (Life Technologies, USA), respectively. The libraries were sequenced at a paired-end 150 bp read length on an Illumina HiseqX Ten. RNA-seq reads were aligned to the reference genome WBcel235 using Tophat v.2.0.6 [60]. The differential gene and transcript expression analysis was performed using Cufflinks tools [61–63]. Genes with a fold change of > 2 (either up or down) and FDR of < 0.01 were considered high temperature-regulated genes. Functional annotation of each gene was analyzed through multiple databases, including Cluster of Orthologous Groups (COG), Gene Ontology (GO), Kyoto Encyclopedia of Genes and Genomes (KEGG), EuKaryotic Orthologous Groups (KOG), Pfam, and evolutionary genealogy of genes: Non-supervised Orthologous Groups (eggNOG). High temperature-regulated genes were further evaluated for functional annotation clustering with Database for Annotation, Visualization, and Integrated Discovery (DAVID) version 6.7 [64]. The RNA sequencing data have been deposited in the GEO with accession number GSE115095.

### ChIP-qPCR

ChIP was performed as previously described with some modifications [65]. Synchronized day 1 adult animals were collected and washed three times with PBS and subjected to three freeze/thaw cycles in liquid nitrogen and then cross-linked with PBS containing 1% formaldehyde for 20 minutes. Worms were then subjected to sonication by using QSonica Q800R2 at 4°C (550 Hz; 30 cycles; 10 s on, 30 s off) in buffer containing 50 mM HEPES pH 7.5, 150 mM NaCl, 1 mM EDTA, 0.1% sodium deoxycholate, 1% Triton X-100, 0.1% SDS, 1 mM PMSF and protease inhibitor cocktail. Cleared lysates were immunoprecipitated using anti-AcH4 (Millipore 06–886), control IgG (Cell signaling 2729) and Salmon sperm DNA/protein A agarose (Millipore 16–157). After washing and elution, DNA was recovered and purified by phenol/chloroform extraction and used for qPCR. The primers used for qPCR can be found in S6 Table.

### Quantification and statistical analysis

Data were presented as mean ± SEM. Survival data were analyzed by using log-rank (Mantel-Cox) test. The levels of fluorescence micrographs were analyzed by using Chi-square and Fisher’s exact tests. Benjamini-Hochberg adjusted p values were used for Figs 1B and 5B and S5E Fig. Two-way ANOVA followed by Bonferroni *post-hoc* test was used for S2A and S4B Figs. Other data were analyzed by using an unpaired Student’s t test. P < 0.05 was considered significant.

## Supporting information

S1 Fig Effects of high environmental temperature on innate immune and detoxification responses. (A) The endogenous *T24B8*.*5* mRNA levels at 15°C and 25°C measured by qPCR. n = 3 for each group. Data are presented as mean ± SEM. ***p < 0.001 via Student’s t test. (B) Expression of *F35E12*.*5p*::GFP upon 25°C cultivation. (C-E) The expression of *T24B8*.*5p*::GFP was induced at 25°C in worms fed with dead *E*. *coli* OP50 killed by UV irradiation (C), heat (D), and kanamycin (E). (F) Pathogen resistance of worms precultivated with UV-killed OP50 at 15°C and 25°C. ***p < 0.001 versus 15°C controls via log-rank test. Scale bar = 100 μm.
(TIF)

S2 Fig PMK-1 is required for gene activation and lifespan regulation at high temperature. (A) Effects of *pmk-1* mutation on defense gene expression at 25°C. n = 3 for each group. Data are presented as mean ± SEM. ***p < 0.001 versus WT controls via two-way ANOVA followed by Bonferroni *post-hoc* test. (B-C) Lifespan of WT and *pmk-1* mutants fed on dead bacteria at 25°C (B) and 15°C (C). ***p < 0.001 versus WT controls via log-rank test.
(TIF)

S3 Fig Persistent stress responses after early life heat exposure. (A-B) Worms were precultivated at 25°C until day 1 adults and transferred to 15°C thereafter. The expression of *F35E12*.*5p*::GFP (A) and *gst-4p*::GFP (B) was examined on day 1, day 4, and day 7. Arrows indicate GFP signals in the posterior of worms. (C) Worms were treated as indicated in S3A and S3B Fig. The mRNA levels of *T24B8*.*5*, *F49F1*.*7* and *cyp-37B1* were examined by qPCR on day 1, day 4, and day 7. n = 3 for each group. Data are presented as mean ± SEM. **p < 0.01, ***p < 0.001 versus 15°C controls via Student’s t test. (D) Persistent resistance to TBHP of worms precultivated at 25°C until day 1 adults. *p < 0.05, **p < 0.01, ***p < 0.001 versus 15°C controls via log-rank test. (E) Persistent mRNA expression of *hsp-16*.*2* in worms exposed to 35°C for one hour on day 1. n = 3 for each group. Data are presented as mean ± SEM. **p < 0.01, ***p < 0.001 versus 20°C controls via Student’s t test. (F) Persistent resistance to heat shock stress of worms exposed to 35°C for one hour on day 1. **p < 0.01, ***p < 0.001 versus 20°C controls via log-rank test. Scale bar = 100 μm.
(TIF)

S4 Fig CBP-1 regulates stress responses at high temperature. (A) Effects of 20-fold diluted *cbp-1* RNAi on the mRNA expression of *cbp-1*. n = 3 for each group. Data are presented as mean ± SEM. ***p < 0.001 via Student’s t test. (B) The effects of *cbp-1* RNAi on the mRNA expression of *T24B8*.*5*, *F49F1*.*7* and *C32H11*.*4* were examined by qPCR at 15°C and 25°C. n = 3 for each group. Data are presented as mean ± SEM. ***p < 0.001 versus control RNAi via two-way ANOVA followed by Bonferroni *post-hoc* test. (C) Effects of RNAi targeting genes regulating histone acetylation on *T24B8*.*5p*::GFP expression at 25°C. HATs: histone acetyltransferases. Number of biological replicates (n): control (95), *cbp-1* RNAi (78), *K03D10*.*3* RNAi (105), *T02C12*.*3* RNAi (85), *ZK856*.*9* RNAi (73), *trr-1* RNAi (64), *ZK1127*.*3* RNAi (102), *mrg-1* RNAi (105). ***p < 0.001 versus control RNAi via Chi-square and Fisher’s exact tests. (D) Effects of *cbp-1* RNAi on *gst-4p*::GFP expression at 25°C. (E) Effects of *cbp-1* RNAi on TBHP resistance at 15°C and 25°C. ***p < 0.001 versus 15°C RNAi via log-rank test. (F-H) Effects of *cbp-1* RNAi on stress gene induction in *eat-2* (F), *daf-2* (G) and *glp-1* (H) mutants. Data are presented as mean ± SEM. *p < 0.05, **p < 0.01, ***p < 0.001 versus control RNAi via Student’s t test. (I) mRNA levels of *T24B8*.*5* measured as indicated in Fig 4E. n = 3 for each group. Data are presented as mean ± SEM. **p < 0.01, ***p < 0.001 versus control RNAi via Student’s t test. Scale bar = 100 μm.
(TIF)

S5 Fig Regulation of histone acetylation by high temperature. (A) Effects of *cbp-1* RNAi on acetylated H4 levels at 15°C and 25°C. (B) Effects of *pmk-1* mutation on acetylated H4 levels at 15°C and 25°C. (C) Schematic of promoter regions amplified by ChIP-qPCR. (D) Acetylated H4 levels on the promoters of *acdh-4* and *pde-4* detected by ChIP-qPCR. n = 3 for each group. Data are presented as mean ± SEM. (E) The expression levels of *acdh-4* and *pde-4* at 15°C and 25°C examined by RNA-seq analysis. n = 3 for each group. Data are presented as mean ± SEM and ns indicates Benjamini-Hochberg adjusted p > 0.05.
(TIF)

S6 Fig SWSN-1 regulates stress responses at high temperature. (A) Effects of *swsn-1* RNAi on *gst-4p*::GFP expression at 25°C. (B) The SWI/SNF complex components in *C*. *elegans*. Asterisks indicate corresponding RNAi clones in *C*. *elegans* Ahringer RNAi collection. (C) TBHP resistance of *swsn-1* RNAi worms precultivated at 15°C and 25°C. ***p < 0.001 versus 15°C RNAi via log-rank test. Scale bar = 100 μm.
(TIF)

S1 Table List of genes regulated by 25°C. Genes with a fold change of > 2 (either up or down) and FDR of < 0.01 were listed as 25°C-regulated genes.
(XLSX)

S2 Table PA14 survival data. (DOCX)

S3 Table TBHP and heat shock resistance survival data. (DOCX)

S4 Table Lifespan data. (DOCX)

S5 Table List of epigenetic genes selected for screen. (DOCX)

S6 Table qPCR primer sequences. (DOCX)
