# Supplementary figures and images for: Histone acetylation promotes long-lasting defense responses and longevity following early life heat stress
Source: PLoS Genet. 2019 Apr 29;15(4):e1008122. doi: 10.1371/journal.pgen.1008122 (PMC6508741; doi:10.1371/journal.pgen.1008122)

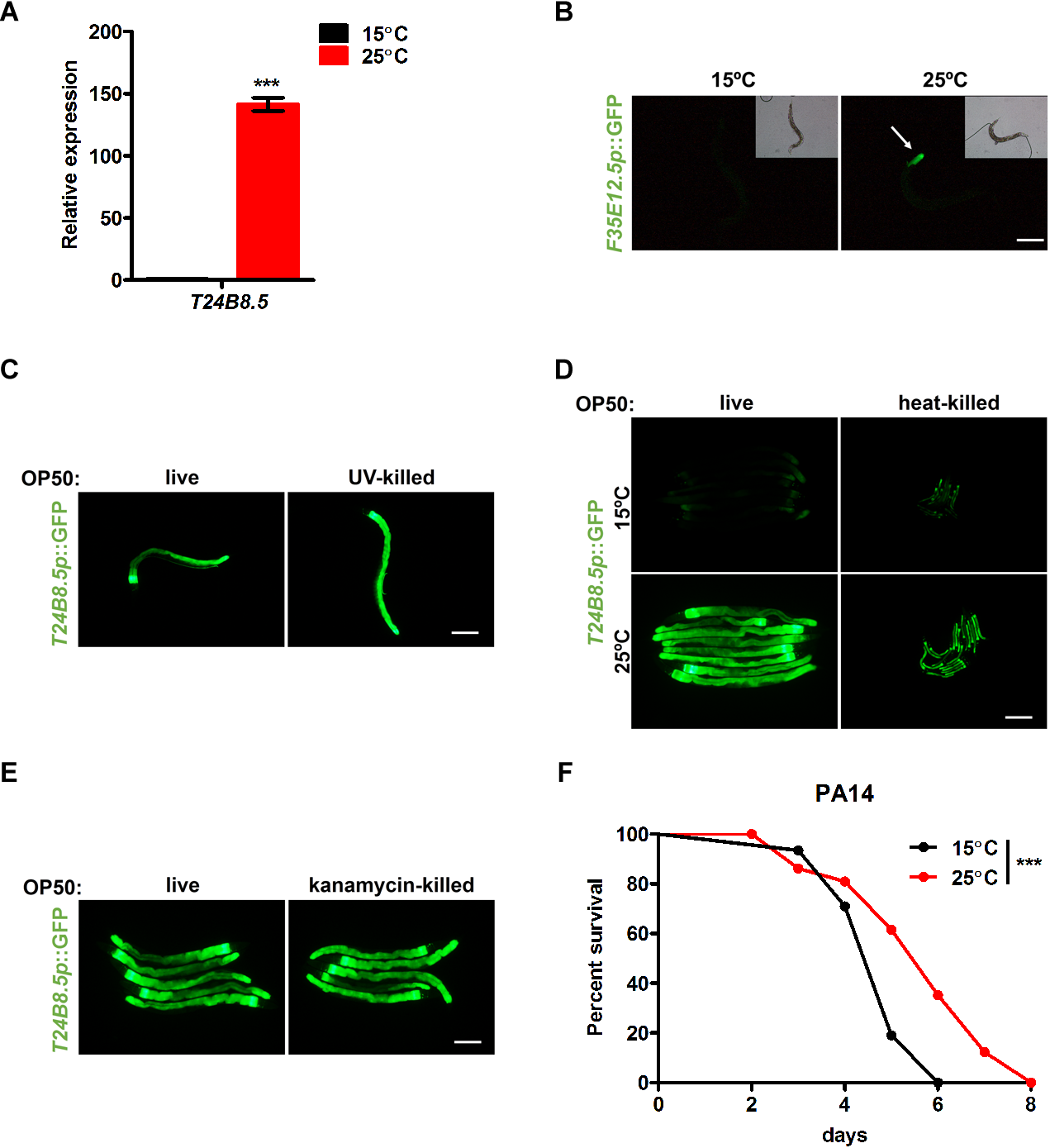

Supplement: S1 Fig — (A) The endogenous T24B8.5 mRNA levels at 15°C and 25°C measured by qPCR. n = 3 for each group. Data are presented as mean ± SEM. ***p < 0.001 via Student’s t test. (B) Expression of F35E12.5p::GFP upon 25°C cultivation. (C-E) The expression of T24B8.5p::GFP was induced at 25°C in worms fed with dead E. coli OP50 killed by UV irradiation (C), heat (D), and kanamycin (E). (F) Pathogen resistance of worms precultivated with UV-killed OP50 at 15°C and 25°C. ***p < 0.001 versus 15°C controls via log-rank test. Scale bar = 100 μm. (TIF) [file pgen.1008122.s001.tif]

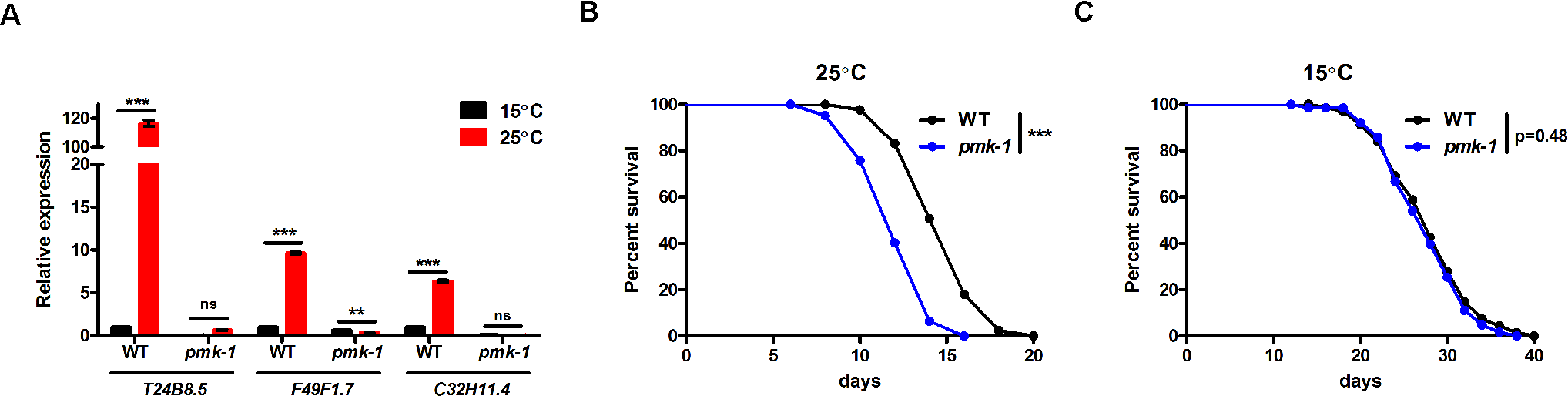

Supplement: S2 Fig — (A) Effects of pmk-1 mutation on defense gene expression at 25°C. n = 3 for each group. Data are presented as mean ± SEM. ***p < 0.001 versus WT controls via two-way ANOVA followed by Bonferroni post-hoc test. (B-C) Lifespan of WT and pmk-1 mutants fed on dead bacteria at 25°C (B) and 15°C (C). ***p < 0.001 versus WT controls via log-rank test. (TIF) [file pgen.1008122.s002.tif]

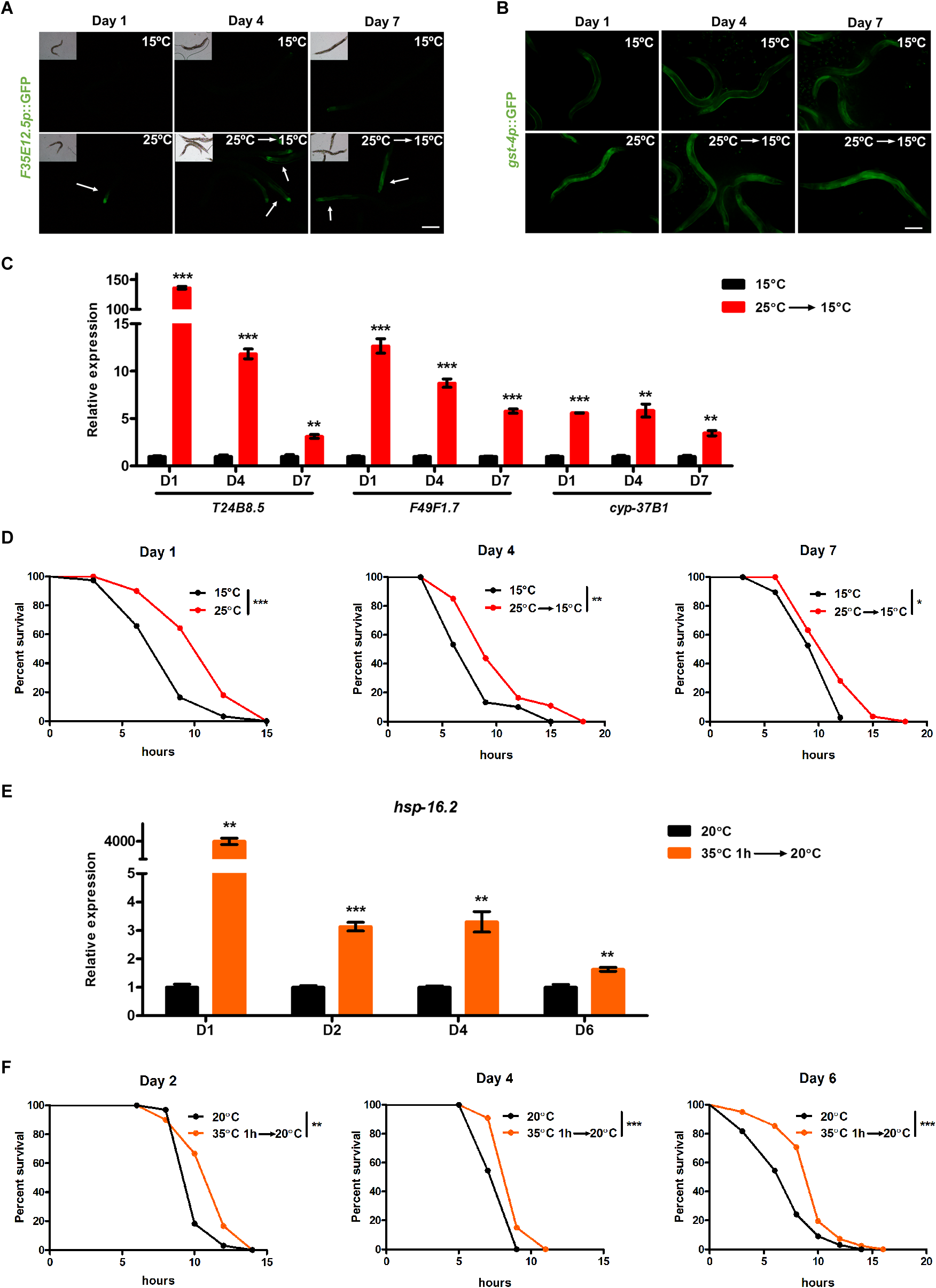

Supplement: S3 Fig — (A-B) Worms were precultivated at 25°C until day 1 adults and transferred to 15°C thereafter. The expression of F35E12.5p::GFP (A) and gst-4p::GFP (B) was examined on day 1, day 4, and day 7. Arrows indicate GFP signals in the posterior of worms. (C) Worms were treated as indicated in S3A and S3B Fig. The mRNA levels of T24B8.5, F49F1.7 and cyp-37B1 were examined by qPCR on day 1, day 4, and day 7. n = 3 for each group. Data are presented as mean ± SEM. **p < 0.01, ***p < 0.001 versus 15°C controls via Student’s t test. (D) Persistent resistance to TBHP of worms precultivated at 25°C until day 1 adults. *p < 0.05, **p < 0.01, ***p < 0.001 versus 15°C controls via log-rank test. (E) Persistent mRNA expression of hsp-16.2 in worms exposed to 35°C for one hour on day 1. n = 3 for each group. Data are presented as mean ± SEM. **p < 0.01, ***p < 0.001 versus 20°C controls via Student’s t test. (F) Persistent resistance to heat shock stress of worms exposed to 35°C for one hour on day 1. **p < 0.01, ***p < 0.001 versus 20°C controls via log-rank test. Scale bar = 100 μm. (TIF) [file pgen.1008122.s003.tif]

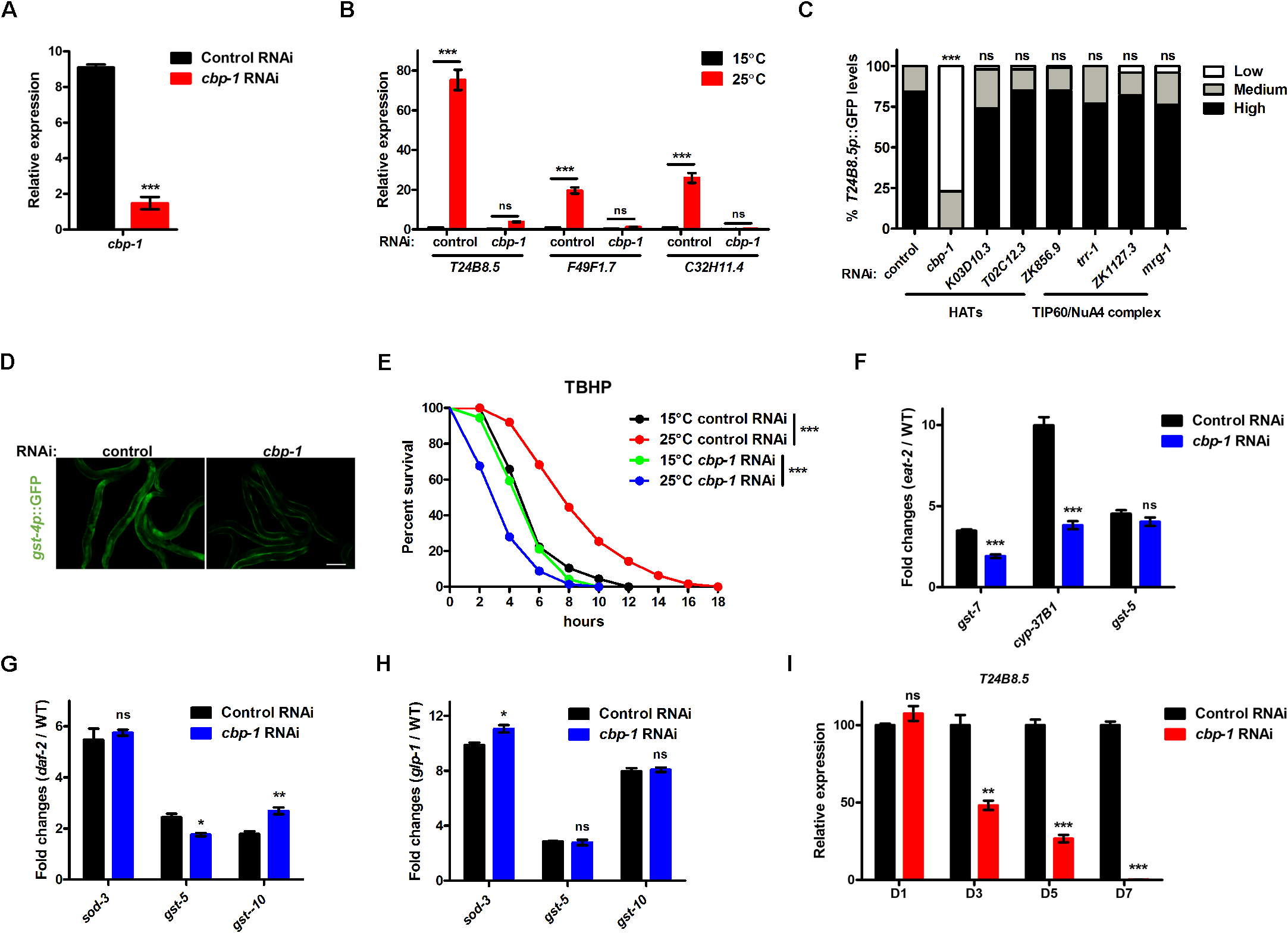

Supplement: S4 Fig — (A) Effects of 20-fold diluted cbp-1 RNAi on the mRNA expression of cbp-1. n = 3 for each group. Data are presented as mean ± SEM. ***p < 0.001 via Student’s t test. (B) The effects of cbp-1 RNAi on the mRNA expression of T24B8.5, F49F1.7 and C32H11.4 were examined by qPCR at 15°C and 25°C. n = 3 for each group. Data are presented as mean ± SEM. ***p < 0.001 versus control RNAi via two-way ANOVA followed by Bonferroni post-hoc test. (C) Effects of RNAi targeting genes regulating histone acetylation on T24B8.5p::GFP expression at 25°C. HATs: histone acetyltransferases. Number of biological replicates (n): control (95), cbp-1 RNAi (78), K03D10.3 RNAi (105), T02C12.3 RNAi (85), ZK856.9 RNAi (73), trr-1 RNAi (64), ZK1127.3 RNAi (102), mrg-1 RNAi (105). ***p < 0.001 versus control RNAi via Chi-square and Fisher’s exact tests. (D) Effects of cbp-1 RNAi on gst-4p::GFP expression at 25°C. (E) Effects of cbp-1 RNAi on TBHP resistance at 15°C and 25°C. ***p < 0.001 versus 15°C RNAi via log-rank test. (F-H) Effects of cbp-1 RNAi on stress gene induction in eat-2 (F), daf-2 (G) and glp-1 (H) mutants. Data are presented as mean ± SEM. *p < 0.05, **p < 0.01, ***p < 0.001 versus control RNAi via Student’s t test. (I) mRNA levels of T24B8.5 measured as indicated in Fig 4E. n = 3 for each group. Data are presented as mean ± SEM. **p < 0.01, ***p < 0.001 versus control RNAi via Student’s t test. Scale bar = 100 μm. (TIF) [file pgen.1008122.s004.tif]

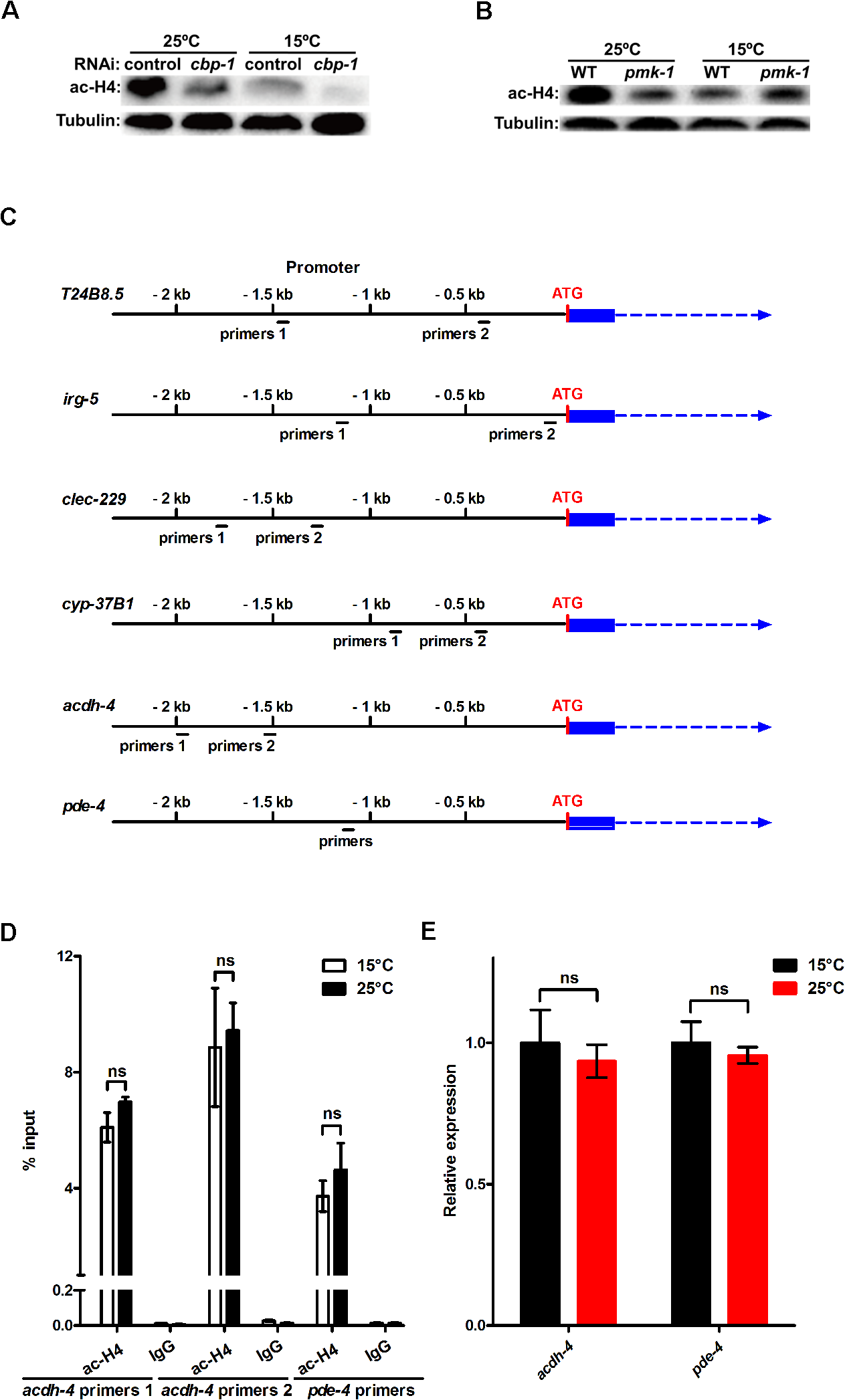

Supplement: S5 Fig — (A) Effects of cbp-1 RNAi on acetylated H4 levels at 15°C and 25°C. (B) Effects of pmk-1 mutation on acetylated H4 levels at 15°C and 25°C. (C) Schematic of promoter regions amplified by ChIP-qPCR. (D) Acetylated H4 levels on the promoters of acdh-4 and pde-4 detected by ChIP-qPCR. n = 3 for each group. Data are presented as mean ± SEM. (E) The expression levels of acdh-4 and pde-4 at 15°C and 25°C examined by RNA-seq analysis. n = 3 for each group. Data are presented as mean ± SEM and ns indicates Benjamini-Hochberg adjusted p > 0.05. (TIF) [file pgen.1008122.s005.tif]

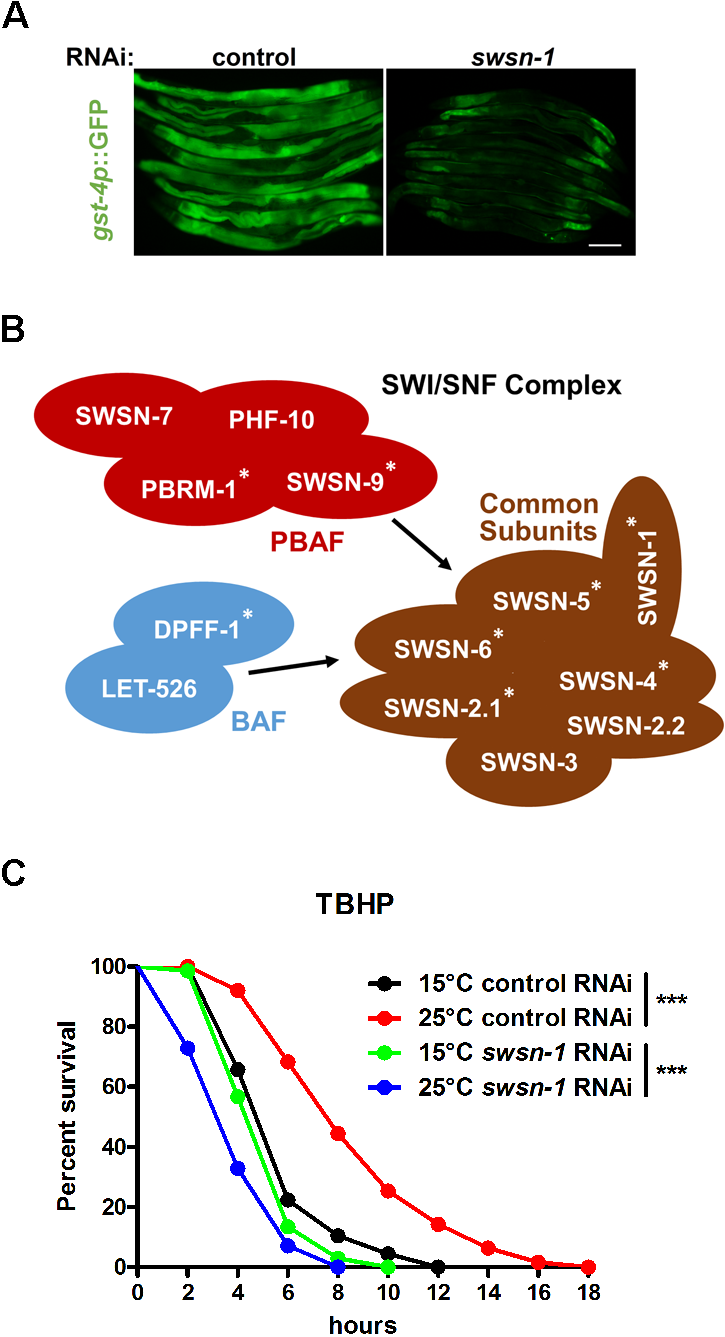

Supplement: S6 Fig — (A) Effects of swsn-1 RNAi on gst-4p::GFP expression at 25°C. (B) The SWI/SNF complex components in C. elegans. Asterisks indicate corresponding RNAi clones in C. elegans Ahringer RNAi collection. (C) TBHP resistance of swsn-1 RNAi worms precultivated at 15°C and 25°C. ***p < 0.001 versus 15°C RNAi via log-rank test. Scale bar = 100 μm. (TIF) [file pgen.1008122.s006.tif]
