# Supplementary material for: Histone acetylation promotes long-lasting defense responses and longevity following early life heat stress
Source: PLoS Genet. 2019 Apr 29;15(4):e1008122. doi: 10.1371/journal.pgen.1008122 (PMC6508741; doi:10.1371/journal.pgen.1008122)
Supplement: S2 Table — (DOCX) [file pgen.1008122.s008.docx]

**S2 Table. PA14 survival data. Repeats 1 are graphed in indicated Figures.**

| Figures | Strain/Treatment | Mean Lifespan  ± SEM (days) | # Worms  Censored/Total | P value |
| --- | --- | --- | --- | --- |
| 1F repeat 1 | 15°C WT | 4.1 ± 0.1 | 3/62 |  |
|  | 20°C WT | 4.8 ± 0.1 | 3/72 | <0.001^a^ |
|  | 25°C WT | 5.3 ± 0.1 | 1/82 | <0.001^a^ |
| 1F repeat 2 | 15°C WT | 4.5 ± 0.1 | 4/46 |  |
|  | 20°C WT | 5.0 ± 0.2 | 2/48 | <0.001^a^ |
|  | 25°C WT | 5.9 ± 0.1 | 4/54 | <0.001^a^ |
| 1F repeat 3 | 15°C WT | 4.4 ± 0.1 | 4/60 |  |
|  | 20°C WT | 5.0 ± 0.1 | 3/50 | <0.001^a^ |
|  | 25°C WT | 6.0 ± 0.2 | 5/59 | <0.001^a^ |
| S1G repeat 1 | 15°C WT on dead OP50 | 4.8 ± 0.1 | 3/61 |  |
|  | 25°C WT on dead OP50 | 5.8 ± 0.2 | 2/59 | <0.001^a^ |
| S1G repeat 2 | 15°C WT on dead OP50 | 4.0 ± 0.1 | 6/49 |  |
|  | 25°C WT on dead OP50 | 5.2 ± 0.1 | 5/53 | <0.001^a^ |
| S1G repeat 3 | 15°C WT on dead OP50 | 4.7 ± 0.1 | 4/53 |  |
|  | 25°C WT on dead OP50 | 6.4 ± 0.1 | 6/63 | <0.001^a^ |
| 2D repeat 1 | 15°C WT | 4.9 ± 0.1 | 3/50 |  |
|  | 25°C WT | 6.0 ± 0.1 | 7/99 | <0.001^a^ |
|  | 15°C *pmk-1* | 3.1 ± 0.1 | 0/48 |  |
|  | 25°C *pmk-1* | 3.2 ± 0.1 | 1/53 | 0.4226^a^ |
| 2D repeat 2 | 15°C WT | 4.4 ± 0.1 | 4/56 |  |
|  | 25°C WT | 5.9 ± 0.2 | 4/65 | <0.001^a^ |
|  | 15°C *pmk-1* | 3.0 ± 0.1 | 0/56 |  |
|  | 25°C *pmk-1* | 3.2 ± 0.1 | 0/55 | 0.2163^a^ |
| 2D repeat 3 | 15°C WT | 5.1 ± 0.1 | 4/49 |  |
|  | 25°C WT | 6.6 ± 0.2 | 6/64 | <0.001^a^ |
|  | 15°C *pmk-1* | 3.1 ± 0.1 | 3/54 |  |
|  | 25°C *pmk-1* | 3.2 ± 0.1 | 2/58 | 0.5044^a^ |
| 3D repeat 1 | 15°C WT day 1 | 3.9 ± 0.1 | 0/50 |  |
|  | 25°C WT day 1 | 5.0 ± 0.1 | 5/65 | <0.001^a^ |
|  | 15°C WT day 4 | 3.2 ± 0.1 | 4/63 |  |
|  | 25°C to 15° at day 1, cultivated at 15° till day 4 | 4.5 ± 0.2 | 2/58 | <0.001^a^ |
|  | 15°C WT day 7 | 1.8 ± 0.1 | 0/57 |  |
|  | 25°C to 15° at day 1, cultivated at 15° till day 7 | 2.7 ± 0.1 | 0/53 | <0.001^a^ |
| 3D repeat 2 | 15°C WT day 1 | 4.6 ± 0.1 | 0/41 |  |
|  | 25°C WT day 1 | 6.4 ± 0.1 | 4/57 | <0.001^a^ |
|  | 15°C WT day 4 | 3.9 ± 0.1 | 4/52 |  |
|  | 25°C to 15° at day 1, cultivated at 15° till day 4 | 5.6 ± 0.2 | 6/56 | <0.001^a^ |
|  | 15°C WT day 7 | 2.7 ± 0.1 | 4/45 |  |
|  | 25°C to 15° at day 1, cultivated at 15° till day 7 | 3.4 ± 0.2 | 1/48 | <0.001^a^ |
| 3D repeat 3 | 15°C WT day 1 | 4.1 ± 0.1 | 1/47 |  |
|  | 25°C WT day 1 | 6.0 ± 0.1 | 1/51 | <0.001^a^ |
|  | 15°C WT day 4 | 3.6 ± 0.1 | 5/51 |  |
|  | 25°C to 15° at day 1, cultivated at 15° till day 4 | 5.3 ± 0.2 | 6/57 | <0.001^a^ |
|  | 15°C WT day 7 | 2.4 ± 0.1 | 3/46 |  |
|  | 25°C to 15° at day 1, cultivated at 15° till day 7 | 3.2 ± 0.2 | 5/55 | <0.001^a^ |
| 4C repeat 1 | 15°C WT control RNAi | 3.7 ± 0.1 | 1/95 |  |
|  | 25°C WT control RNAi | 4.8 ± 0.1 | 1/93 | <0.001^a^ |
|  | 15°C WT *cbp-1* RNAi | 3.0 ± 0.04 | 1/72 |  |
|  | 25°C WT *cbp-1* RNAi | 2.8 ± 0.1 | 1/74 | <0.001^a^ |
| 4C repeat 2 | 15°C WT control RNAi | 3.9 ± 0.1 | 6/88 |  |
|  | 25°C WT control RNAi | 4.6 ± 0.1 | 4/75 | <0.001^a^ |
|  | 15°C WT *cbp-1* RNAi | 2.9 ± 0.1 | 5/84 |  |
|  | 25°C WT *cbp-1* RNAi | 2.7 ± 0.1 | 5/91 | <0.001^a^ |
| 4C repeat 3 | 15°C WT control RNAi | 3.9 ± 0.1 | 6/88 |  |
|  | 25°C WT control RNAi | 4.8 ± 0.1 | 4/75 | <0.001^a^ |
|  | 15°C WT *cbp-1* RNAi | 3.0 ± 0.1 | 5/84 |  |
|  | 25°C WT *cbp-1* RNAi | 2.8 ± 0.1 | 5/91 | <0.001^a^ |
| 6D repeat 1 | 15°C WT control RNAi | 2.8 ± 0.1 | 1/73 |  |
|  | 25°C WT control RNAi | 3.9 ± 0.2 | 2/73 | <0.001^a^ |
|  | 15°C WT *swsn-1* RNAi | 2.5 ± 0.1 | 1/76 |  |
|  | 25°C WT *swsn-1* RNAi | 2.3 ± 0.1 | 2/73 | 0.1837^a^ |
| 6D repeat 2 | 15°C WT control RNAi | 3.1 ± 0.1 | 6/72 |  |
|  | 25°C WT control RNAi | 5.3 ± 0.2 | 4/117 | <0.001^a^ |
|  | 15°C WT *swsn-1* RNAi | 3.0 ± 0.1 | 3/100 |  |
|  | 25°C WT *swsn-1* RNAi | 3.0 ± 0.1 | 2/101 | 0.3771^a^ |
| 6D repeat 3 | 15°C WT control RNAi | 3.0 ± 0.1 | 3/73 |  |
|  | 25°C WT control RNAi | 5.0 ± 0.2 | 7/109 | <0.001^a^ |
|  | 15°C WT *swsn-1* RNAi | 2.7 ± 0.1 | 4/103 |  |
|  | 25°C WT *swsn-1* RNAi | 2.6 ± 0.1 | 2/94 | 0.3552^a^ |

^a^ vs same treatment/strain at 15°C
