# Supplementary material for: Histone acetylation promotes long-lasting defense responses and longevity following early life heat stress
Source: PLoS Genet. 2019 Apr 29;15(4):e1008122. doi: 10.1371/journal.pgen.1008122 (PMC6508741; doi:10.1371/journal.pgen.1008122)
Supplement: S3 Table — (DOCX) [file pgen.1008122.s009.docx]

**S3 Table. TBHP and heat shock resistance survival data. Repeats 1 are graphed in indicated Figures.**

| Figures | Strain/Treatment | Mean Lifespan  ± SEM (hours) | # Worms  Censored/Total | P value |
| --- | --- | --- | --- | --- |
| 1G repeat 1 | 15°C WT | 8.3 ± 0.3 | 1/36 |  |
|  | 25°C WT | 13.4 ± 0.5 | 2/36 | <0.001^a^ |
| 1G repeat 2 | 15°C WT | 8.6 ± 0.3 | 1/40 |  |
|  | 25°C WT | 11.7 ± 0.5 | 0/45 | <0.001^a^ |
| 1G repeat 3 | 15°C WT | 8.1 ± 0.3 | 2/37 |  |
|  | 25°C WT | 12.4 ± 0.4 | 0/41 | <0.001^a^ |
| S3D repeat 1 | 15°C WT day 1 | 8.5 ± 0.4 | 2/38 |  |
|  | 25°C WT day 1 | 11.2 ± 0.4 | 1/40 | <0.001^a^ |
|  | 15°C WT day 4 | 8.3 ± 0.5 | 1/31 |  |
|  | 25°C to 15° at day 1, cultivated at 15° till day 4 | 10.7 ± 0.6 | 4/41 | 0.0024^a^ |
|  | 15°C WT day 7 | 10.3 ± 0.3 | 3/39 |  |
|  | 25°C to 15° at day 1, cultivated at 15° till day 7 | 11.9 ± 0.5 | 3/33 | 0.015^a^ |
| S3D repeat 2 | 15°C WT day 1 | 8.7 ± 0.4 | 2/42 |  |
|  | 25°C WT day 1 | 12.0 ± 0.4 | 3/51 | <0.001^a^ |
|  | 15°C WT day 4 | 8.3 ± 0.4 | 1/38 |  |
|  | 25°C to 15° at day 1, cultivated at 15° till day 4 | 10.7 ± 0.5 | 1/45 | <0.001^a^ |
|  | 15°C WT day 7 | 8.2 ± 0.3 | 2/40 |  |
|  | 25°C to 15° at day 1, cultivated at 15° till day 7 | 9.9 ± 0.5 | 0/42 | 0.003^a^ |
| S3D repeat 3 | 15°C WT day 1 | 8.3 ± 0.3 | 4/45 |  |
|  | 25°C WT day 1 | 12.8 ± 0.4 | 4/39 | <0.001^a^ |
|  | 15°C WT day 4 | 7.9 ± 0.3 | 0/48 |  |
|  | 25°C to 15° at day 1, cultivated at 15° till day 4 | 9.3 ± 0.4 | 1/40 | 0.009^a^ |
|  | 15°C WT day 7 | 8.1 ± 0.4 | 3/41 |  |
|  | 25°C to 15° at day 1, cultivated at 15° till day 7 | 9.4 ± 0.5 | 0/41 | 0.0288^a^ |
| S3F repeat 1 | 20°C WT day 2 | 10.4 ± 0.2 | 1/34 |  |
|  | 35°C 1h at day 1, cultivated at 20° till day 2 | 11.5 ± 0.3 | 1/31 | 0.0016^b^ |
|  | 20°C WT day 4 | 8.1 ± 0.2 | 1/34 |  |
|  | 35°C 1h at day 1, cultivated at 20° till day 4 | 9.1 ± 0.2 | 1/34 | <0.001^b^ |
|  | 20°C WT day 6 | 7.3 ± 0.5 | 0/33 |  |
|  | 35°C 1h at day 1, cultivated at 20° till day 6 | 9.5 ± 0.4 | 0/41 | <0.001^b^ |
| S3F repeat 2 | 20°C WT day 2 | 6.9 ± 0.1 | 0/34 |  |
|  | 35°C 1h at day 1, cultivated at 20° till day 2 | 7.9 ± 0.2 | 1/36 | <0.001^b^ |
|  | 20°C WT day 4 | 7.2 ± 0.3 | 0/31 |  |
|  | 35°C 1h at day 1, cultivated at 20° till day 4 | 9.7 ± 0.2 | 1/41 | <0.001^b^ |
|  | 20°C WT day 6 | 6.8 ± 0.2 | 0/32 |  |
|  | 35°C 1h at day 1, cultivated at 20° till day 6 | 9.0 ± 0.4 | 1/36 | <0.001^b^ |
| S3F repeat 3 | 20°C WT day 2 | 7.7 ± 0.2 | 0/34 |  |
|  | 35°C 1h at day 1, cultivated at 20° till day 2 | 11.7 ± 0.3 | 1/31 | <0.001^b^ |
|  | 20°C WT day 4 | 6.9 ± 0.2 | 1/37 |  |
|  | 35°C 1h at day 1, cultivated at 20° till day 4 | 9.9 ± 0.2 | 2/31 | <0.001^b^ |
|  | 20°C WT day 6 | 7.9 ± 0.2 | 0/40 |  |
|  | 35°C 1h at day 1, cultivated at 20° till day 6 | 9.6 ± 0.3 | 1/51 | <0.001^b^ |
| S4E repeat 1 | 15°C WT control RNAi | 6.1 ± 0.3 | 2/69 |  |
|  | 25°C WT control RNAi | 9.1 ± 0.4 | 7/70 | <0.001^a^ |
|  | 15°C WT *cbp-1* RNAi | 5.6 ± 0.2 | 2/73 |  |
|  | 25°C WT *cbp-1* RNAi | 4.1 ± 0.2 | 0/68 | <0.001^a^ |
| S4E repeat 2 | 15°C WT control RNAi | 8.8 ± 0.5 | 3/79 |  |
|  | 25°C WT control RNAi | 11.8 ± 0.4 | 4/75 | <0.001^a^ |
|  | 15°C WT *cbp-1* RNAi | 5.6 ± 0.2 | 2/65 |  |
|  | 25°C WT *cbp-1* RNAi | 4.6 ± 0.2 | 3/73 | 0.003^a^ |
| S4E repeat 3 | 15°C WT control RNAi | 11.1 ± 0.5 | 4/64 |  |
|  | 25°C WT control RNAi | 14.5 ± 0.5 | 2/76 | <0.001^a^ |
|  | 15°C WT *cbp-1* RNAi | 9.4 ± 0.5 | 3/65 |  |
|  | 25°C WT *cbp-1* RNAi | 4.4 ± 0.3 | 4/72 | <0.001^a^ |
| S6C repeat 1 | 15°C WT control RNAi | 6.1 ± 0.3 | 2/69 |  |
|  | 25°C WT control RNAi | 9.1 ± 0.4 | 7/70 | <0.001^a^ |
|  | 15°C WT *swsn-1* RNAi | 5.3 ± 0.2 | 2/67 |  |
|  | 25°C WT *swsn-1* RNAi | 4.3 ± 0.2 | 0/70 | <0.001^a^ |
| S6C repeat 2 | 15°C WT control RNAi | 6.4 ± 0.4 | 5/65 |  |
|  | 25°C WT control RNAi | 10.7 ± 0.6 | 3/69 | <0.001^a^ |
|  | 15°C WT *swsn-1* RNAi | 5.3 ± 0.3 | 5/75 |  |
|  | 25°C WT *swsn-1* RNAi | 3.8 ± 0.2 | 2/69 | <0.001^a^ |
| S6C repeat 3 | 15°C WT control RNAi | 6.3 ± 0.3 | 4/68 |  |
|  | 25°C WT control RNAi | 10.3 ± 0.5 | 3/68 | <0.001^a^ |
|  | 15°C WT *swsn-1* RNAi | 5.3 ± 0.2 | 3/69 |  |
|  | 25°C WT *swsn-1* RNAi | 4.0 ± 0.2 | 1/68 | <0.001^a^ |

^a^ vs same treatment/strain at 15°C

^b^ vs same strain at 20°C
