# Supplementary material for: Histone acetylation promotes long-lasting defense responses and longevity following early life heat stress
Source: PLoS Genet. 2019 Apr 29;15(4):e1008122. doi: 10.1371/journal.pgen.1008122 (PMC6508741; doi:10.1371/journal.pgen.1008122)
Supplement: S4 Table — (DOCX) [file pgen.1008122.s010.docx]

**S4 Table. Lifespan data. Repeats 1 are graphed in indicated Figures.**

| Figures | Strain/Treatment | Mean Lifespan  ± SEM (days) | # Worms  Censored/Total | P value |
| --- | --- | --- | --- | --- |
| 2E repeat 1 | 25°C WT | 12.8 ± 0.3 | 7/63 |  |
|  | 25°C *pmk-1* | 10.5 ± 0.5 | 5/50 | <0.001^a^ |
| 2E repeat 2 | 25°C WT | 13.0 ± 0.3 | 7/64 |  |
|  | 25°C *pmk-1* | 10.8 ± 0.3 | 5/66 | <0.001^a^ |
| 2E repeat 3 | 25°C WT | 13.5 ± 0.4 | 4/67 |  |
|  | 25°C *pmk-1* | 10.5 ± 0.2 | 7/64 | <0.001^a^ |
| 2F repeat 1 | 15°C WT | 28.2 ± 0.7 | 5/98 |  |
|  | 15°C *pmk-1* | 29.3 ± 0.6 | 7/115 | 0.3869^a^ |
| 2F repeat 2 | 15°C WT | 24.9 ± 0.6 | 8/99 |  |
|  | 15°C *pmk-1* | 24.1 ± 0.7 | 7/98 | 0.4861^a^ |
| 2F repeat 3 | 15°C WT | 24.6 ± 0.7 | 8/114 |  |
|  | 15°C *pmk-1* | 24.4 ± 0.6 | 8/104 | 0.5161^a^ |
| S2B repeat 1 | 25°C WT on dead OP50 | 15.0 ± 0.2 | 3/86 |  |
|  | 25°C *pmk-1* on dead OP50 | 12.4 ± 0.3 | 3/65 | <0.001^a^ |
| S2B repeat 2 | 25°C WT on dead OP50 | 14.9 ± 0.2 | 4/85 |  |
|  | 25°C *pmk-1* on dead OP50 | 12.5 ± 0.3 | 2/74 | <0.001^a^ |
| S2B repeat 3 | 25°C WT on dead OP50 | 15 ± 0.2 | 2/83 |  |
|  | 25°C *pmk-1* on dead OP50 | 12.3 ± 0.3 | 3/65 | <0.001^a^ |
| S2C repeat 1 | 15°C WT on dead OP50 | 27.9 ± 0.6 | 2/70 |  |
|  | 15°C *pmk-1* on dead OP50 | 27.5 ± 0.6 | 3/66 | 0.4839^a^ |
| S2C repeat 2 | 15°C WT on dead OP50 | 27.8 ± 0.6 | 4/78 |  |
|  | 15°C *pmk-1* on dead OP50 | 27.4 ± 0.6 | 6/72 | 0.5097^a^ |
| S2C repeat 3 | 15°C WT on dead OP50 | 27.4 ± 0.6 | 4/69 |  |
|  | 15°C *pmk-1* on dead OP50 | 27.0 ± 0.6 | 4/69 | 0.6512^a^ |
| 7A repeat 1 | 15°C WT control RNAi | 22.6 ± 0.5 | 5/130 |  |
|  | 25°C to 15°C at L4, WT control RNAi | 26.5 ± 0.6 | 6/111 | <0.001^b^ |
| 7A repeat 2 | 15°C WT control RNAi | 24.4 ± 0.9 | 4/45 |  |
|  | 25°C to 15°C at L4, WT control RNAi | 29.8 ± 0.7 | 6/104 | <0.001^b^ |
| 7A repeat 3 | 15°C WT control RNAi | 22.4 ± 0.6 | 4/91 |  |
|  | 25°C to 15°C at L4, WT control RNAi | 26.6 ± 0.6 | 5/110 | <0.001^b^ |
| 7B repeat 1 | 15°C WT *cbp-1* RNAi | 11.4 ± 0.2 | 1/142 |  |
|  | 25°C to 15°C at L4, WT *cbp-1* RNAi | 8.8 ± 0.2 | 0/120 | <0.001^b^ |
| 7B repeat 2 | 15°C WT *cbp-1* RNAi | 15.8 ± 0.3 | 3/95 |  |
|  | 25°C to 15°C at L4, WT *cbp-1* RNAi | 13.4 ± 0.3 | 3/139 | <0.001^b^ |
| 7B repeat 3 | 15°C WT *cbp-1* RNAi | 12.8 ± 0.3 | 2/120 |  |
|  | 25°C to 15°C at L4, WT *cbp-1* RNAi | 10.4 ± 0.3 | 3/112 | <0.001^b^ |
| 7C repeat 1 | 15°C WT *swsn-1* RNAi | 14.4 ± 0.6 | 3/72 |  |
|  | 25°C to 15°C at L4, WT *swsn-1* RNAi | 15.8 ± 0.4 | 3/63 | 0.3267^b^ |
| 7C repeat 2 | 15°C WT *swsn-1* RNAi | 15.0 ± 0.3 | 9/133 |  |
|  | 25°C to 15°C at L4, WT *swsn-1* RNAi | 16.0 ± 0.3 | 6/129 | 0.0817^b^ |
| 7C repeat 3 | 15°C WT *swsn-1* RNAi | 13.7 ± 0.3 | 7/129 |  |
|  | 25°C to 15°C at L4, WT *swsn-1* RNAi | 14.9 ± 0.3 | 7/130 | 0.015^b^ |
| 7D repeat 1 | 15°C *pmk-1* | 23.3 ± 0.8 | 7/67 |  |
|  | 25°C to 15°C at L4, *pmk-1* | 23.5 ± 0.8 | 5/53 | 0.6393^b^ |
| 7D repeat 2 | 15°C *pmk-1* | 22.6 ± 1.0 | 5/58 |  |
|  | 25°C to 15°C at L4, *pmk-1* | 24.6 ± 0.8 | 4/80 | 0.2322^b^ |
| 7D repeat 3 | 15°C *pmk-1* | 23.2 ± 0.9 | 5/65 |  |
|  | 25°C to 15°C at L4, *pmk-1* | 24.0 ± 0.8 | 4/69 | 0.7295^b^ |

^a^ vs WT controls at same temperature

^b^ vs same treatment/strain at 15°C
