# Supplementary material for: Histone acetylation promotes long-lasting defense responses and longevity following early life heat stress
Source: PLoS Genet. 2019 Apr 29;15(4):e1008122. doi: 10.1371/journal.pgen.1008122 (PMC6508741; doi:10.1371/journal.pgen.1008122)
Supplement: S5 Table — (DOCX) [file pgen.1008122.s011.docx]

**S5 Table. List of epigenetic genes selected for screen.**

| Histone acetyltransferase genes |
| --- |
| *cbp-1*, *K03D10.3*, *T02C12.3*, *ZK856.9, trr-1*, *ZK1127.3*, *mrg-1* |
| Histone deacetylase genes |
| *hda-1*, *hda-2*, *hda-3*, *hda-4*, *hda-11* |
| Histone methyltrasferase genes |
| *set-1*, *set-2*, *set-3*, *set-4*, *set-5*, *set-6*, *set-8*, *set-9*, *set-10*, *set-11*, *set-12*, *set-14*, *set-15*, *set-16*, *set-17*, *set-18*, *set-19*, *set-20*, *set-25*, *set-26*, *set-30*, *set-32*, *lin-59*, *ttll-12*, *blmp-1, ash-2*, *rha-1* |
| Histone demethylase genes |
| *jmjc-1*, *jmjd-1.1*, *jmjd-1.2*, *jmjd-2*, *jmjd-3.1*, *jmjd-3.2*, *jmjd-3.3*, *jmjd-5*, *jhdm-1*, *rbr-2*, *psr-1*, *utx-1*, *amx-1*, *amx-3*, *hpo-15*, *spr-5*, *lsd-1*, *vps-22*, *F55C5.6* |
| Chromatin remodeling genes |
| *hsp-1*, *swsn-1*, swsn-4, *snfc-5*, ssl-1, swsn-2.1, *swsn-6*, *l*in-53, mep-1, isw-1, pyp-1, rba-1, *arp-6*, C17E4.6, *zhit-1*, gfl-1, *htz-1*, *ssl-1* |
