# Supplementary material for: Histone acetylation promotes long-lasting defense responses and longevity following early life heat stress
Source: PLoS Genet. 2019 Apr 29;15(4):e1008122. doi: 10.1371/journal.pgen.1008122 (PMC6508741; doi:10.1371/journal.pgen.1008122)
Supplement: S6 Table — (DOCX) [file pgen.1008122.s012.docx]

**S6 Table. qPCR primer sequences**

| Primers | Sequences |
| --- | --- |
| *snb-1* Forward | GCAAGTATTGGTGGAAGA |
| *snb-1* Reverse | ACGATGATGATAATAAGAATGAC |
| *T24B8.5* Forward | TTGTGATTGTGCTTGTAG |
| *T24B8.5* Reverse | CAACCACTTCTAACATCTG |
| *gst-4* Forward | GGCAATAATCAATAAGTTCCT |
| *gst-4* Reverse | GTCAGCAATCACAATATCAG |
| *gst-5* Forward | TTCCTACAAGTTGACCTA |
| *gst-5* Reverse | TGACTCTATTATCCTCGTAT |
| *gst-7* Forward | AGGATTCAAGAAGCACTATG |
| *gst-7* Reverse | AAGAGTCTCCAACCAAGTA |
| *gst-10* Forward | CATCCGTCTTCTGTTCCT |
| *gst-10* Reverse | AACTCTTGCCATTCATTCC |
| *gst-38* Forward | TTACACAAGCGTCTATCT |
| *gst-38* Reverse | AGCAACAAGCAAATCAAT |
| *cbp-1* Forward | CATTCCAAAACGGACCAAAT |
| *cbp-1* Reverse | GCTTCCTGATTCGGTTCC |
| *mtl-1* Forward | ATGGCTTGCAAGTGTGACTG |
| *mtl-1* Reverse | CACATTTGTCTCCGCACTTG |
| *sod-3* Forward | CCAACCAGCGCTGAAATTCAATGG |
| *sod-3* Reverse | GGAACCGAAGTCGCGCTTAATAGT |
| *ins-7* Forward | TCGTTGTGGAAGAAGAATACATTC |
| *ins -7* Reverse | TTAAGGACAGCACTGTTTTCG |
| *hsp-16.1* Forward | GCAGAGGCTCTCCATCTGAA |
| *hsp-16.1* Reverse | GCTTGAACTGCGAGACATTG |
| *hsp-16.2* Forward | ACCTGAAGATGTAGATGTTG |
| *hsp-16.2* Reverse | TTGCCTGTTGAATTGGAA |
| *dod-24* Forward | TGTCCAACACAACCTGCATT |
| *dod-24* Reverse | TGTGTCCCGAGTAACAACCA |
| *C32H11.4* Forward | CTCTCCTGGCTTCTGTAG |
| *C32H11.4* Reverse | CGATTACTGGCTTGTGAAC |
| *dod-22* Forward | TTATAGTAGAGGTGGAATAC |
| *dod-22* Reverse | CACTTGAGAATGAATACG |
| *dod-17* Forward | GTTGGATTGTGACAGTTC |
| *dod-17* Reverse | TTGAGTAGTCCGAATAAGAT |
| *cyp-37B1* Forward | GATGGACTCTATGGTGTT |
| *cyp-37B1* Reverse | ACTGACGGATACATTCTTA |
| *F49F1.5* Forward | ATGCCTCATTGTTCTATT |
| *F49F1.5* Reverse | CTATACGGACATTCACTT |
| *mul-1* Forward | CATACACCTGTGGAACCT |
| *mul-1* Reverse | TATAATTTGGGCACTCAATCAT |
| *F49F1.7* Forward | CTACAGGCTACACAGACT |
| *F49F1.7* Reverse | CGGTAGTTGATGGAGTTG |
| *cyp-37B1* ChIP-qPCR primers 1 Forward | TCTACCAACTATGCTTGTCA |
| *cyp-37B1* ChIP-qPCR primers 1 Reverse | TCTGATCTCACATCGTTCTT |
| *cyp-37B1* ChIP-qPCR primers 2 Forward | CCTGCTCATTGTGTTACC |
| *cyp-37B1* ChIP-qPCR primers 2 Reverse | GTATAAGGGGCTCAACTCA |
| *T24B8.5* ChIP-qPCR primers 1 Forward | CCAGTCCGTACATCTTCT |
| *T24B8.5* ChIP-qPCR primers 1 Reverse | TTCAATATTTCACACACTTCG |
| *T24B8.5* ChIP-qPCR primers 2 Forward | GAATGCTTCTGGTTTGTTAC |
| *T24B8.5* ChIP-qPCR primers 2 Reverse | ATGTCTCTCTGCTCCAAA |
| *irg-5* ChIP-qPCR primers 1 Forward | AGGTTGTACCTGTTTCCGCT |
| *irg-5* ChIP-qPCR primers 1 Reverse | ACATTGAAATCAGTTCAGGGTGA |
| *irg-5* ChIP-qPCR primers 2 Forward | ATAAAAGGAGCGACGGACAC |
| *irg-5* ChIP-qPCR primers 2 Reverse | TGAAAGAAAATGCTTGCAGTGA |
| *clec-229* ChIP-qPCR primers 1 Forward | GGATACGCTCCTTATGCACC |
| *clec-229* ChIP-qPCR primers 1 Reverse | GGGAAGCAGTAATGAAGCTCT |
| *clec-229* ChIP-qPCR primers 2 Forward | TCAACCAGAGGTACGGTCA |
| *clec-229* ChIP-qPCR primers 2 Reverse | GAGAGAAGTCATTGGGGCAT |
| *acdh-4* ChIP-qPCR primers 1 Forward | TTCGCAAAACAGTTATATTTGAACC |
| *acdh-4* ChIP-qPCR primers 1 Reverse | ATCGCACTGAAAACAAATCGG |
| *acdh-4* ChIP-qPCR primers 2 Forward | AAAGAGGAAAGCATTGAAACAGAT |
| *acdh-4* ChIP-qPCR primers 2 Reverse | TGTTCTGGATCCTCAAAAGTCTC |
| *pde-4*  ChIP-qPCR Forward | ACCTCCTCCCTTTTTCTGCTA |
| *pde-4*  ChIP-qPCR Forward | CGAATCGGGGTGAAAAGACG |
